# Cell-type-specific CAG repeat expansions and toxicity of mutant Huntingtin in human striatum and cerebellum

**DOI:** 10.1038/s41588-024-01653-6

**Published:** 2024-01-30

**Authors:** Kert Mätlik, Matthew Baffuto, Laura Kus, Amit Laxmikant Deshmukh, David A. Davis, Matthew R. Paul, Thomas S. Carroll, Marie-Christine Caron, Jean-Yves Masson, Christopher E. Pearson, Nathaniel Heintz

**Affiliations:** 1 Laboratory of Molecular Biology, The Rockefeller University, New York, NY USA; 2 Program of Genetics & Genome Biology, The Hospital for Sick Children, Toronto, Ontario Canada; 3 Miller School of Medicine, University of Miami, Miami, FL USA; 4 Bioinformatics Resource Center, The Rockefeller University, New York, NY USA; 5 CHU de Québec Research Center, Oncology Division, Laval University Cancer Research Center, Quebec City, Quebec Canada; 6 Program of Molecular Genetics, University of Toronto, Toronto, Ontario Canada

**Keywords:** Movement disorders, Neurodegenerative diseases

## Abstract

Brain region-specific degeneration and somatic expansions of the mutant Huntingtin (*mHTT*) CAG tract are key features of Huntington’s disease (HD). However, the relationships among CAG expansions, death of specific cell types and molecular events associated with these processes are not established. Here, we used fluorescence-activated nuclear sorting (FANS) and deep molecular profiling to gain insight into the properties of cell types of the human striatum and cerebellum in HD and control donors. CAG expansions arise at *mHTT* in striatal medium spiny neurons (MSNs), cholinergic interneurons and cerebellar Purkinje neurons, and at mutant *ATXN3* in MSNs from SCA3 donors. CAG expansions in MSNs are associated with higher levels of MSH2 and MSH3 (forming MutSβ), which can inhibit nucleolytic excision of CAG slip-outs by FAN1. Our data support a model in which CAG expansions are necessary but may not be sufficient for cell death and identify transcriptional changes associated with somatic CAG expansions and striatal toxicity.

## Abstract (web summary)

Fluorescence-activated nuclear sorting combined with deep profiling shows that Huntington’s disease repeat expansions arise in specific cell types and are associated with elevated MSH2 and MSH3, which promote expansions in vitro by inhibiting excision of CAG slip-outs by FAN1.

## Main

Huntington’s disease (HD) is a fatal late-onset neurodegenerative disease caused by an abnormally long CAG tract in exon 1 of the Huntingtin gene (*HTT*)^1^. HD age at onset is most often defined as the onset of motor symptoms, which are thought to arise as a consequence of early degeneration of the caudate nucleus and putamen, primarily due to loss of projection neurons of these structures, known as medium-sized spiny neurons of the direct and indirect pathways (dMSNs and iMSNs)^2^. Remarkably, other neuron types within these same structures are largely spared from cell death^3–5^. However, *HTT* is ubiquitously expressed, and the reason for selective vulnerability of specific cell types in HD is largely unknown^6^.

Tissue-specific ongoing CAG repeat expansions of the mutant allele are a central feature of HD and other repeat expansion disorders^7–11^. Expansion of the inherited *mHTT* allele to very long CAG tracts has been observed sporadically in various brain structures, including the caudate nucleus and putamen, but not in the cerebellum^12^. A causal role for somatic expansions of the CAG repeat in HD pathogenesis is supported by findings from a genome-wide association study looking for genetic modifiers of HD motor symptom onset other than CAG tract length itself^13,14^. Although analysis of individual cells captured from HD striatum and cortex by laser-microdissection capture has indicated that somatic expansion occurs more frequently in neurons^15^, it is not known whether CAG expansions occur in specific types of neuronal and glial cells in these regions. Therefore, it is unclear if CAG expansions are sufficient to explain selective cellular vulnerability in HD and what cell-specific factors in addition to somatically expanded *mHTT* CAG tract are required for toxicity.

To gain further insight into somatic CAG expansion and toxicity in HD, we developed fluorescence-activated nuclear sorting (FANS) methods for isolation of large numbers of nuclei from human striatal cell types, and we examined the relationships among selective cellular vulnerability, somatic CAG expansion and transcriptional responses in HD. We find that extensive somatic expansion of the *mHTT* CAG tract occurs in both medium spiny neuron (MSN) populations that are selectively vulnerable in HD, as well as in cholinergic interneurons that are not lost in the HD striatum, although we cannot rule out cell-type-specific somatic CAG expansions beyond the length limit of our assay. CAG expansion is observed also at the mutant *ATXN3* locus in MSN nuclei isolated from the post-mortem brains of spinocerebellar ataxia 3 (SCA3) donors, indicating that MSNs are intrinsically prone to somatic expansion of CAG tracts. We demonstrate that the levels of DNA mismatch repair (MMR) proteins MSH2 and MSH3 are elevated in MSN nuclei, suggesting that these proteins may contribute to preferential somatic CAG expansions in MSNs. We offer mechanistic insight into how MutSβ could be promoting somatic CAG expansions by showing that increased concentrations of MutSβ inhibit excision rates of excess slipped-CAG repeats, putative intermediates of expansion mutations, by FAN1 nuclease. Our findings support models in which somatic expansion of the *mHTT* CAG tract is a critical first step in HD pathogenesis^16^, and they identify specific genes whose altered expression may modulate toxicity in HD.

## Results

### FANS-seq profiling of human striatal cell types

**Fig. 1:**
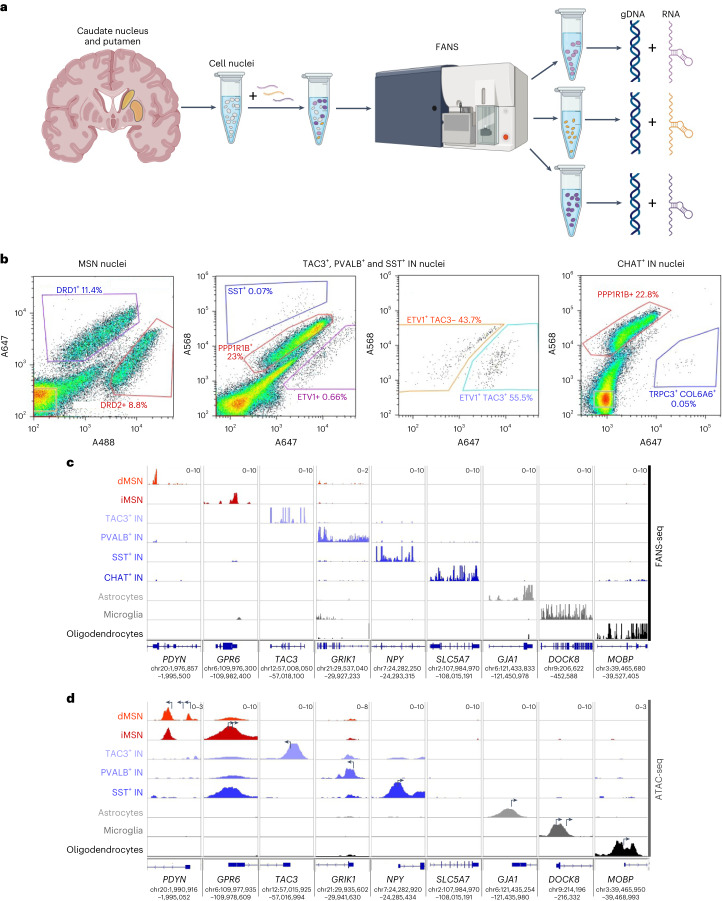
FANS-based isolation of nuclei of striatal cell types from human post-mortem caudate nucleus and putamen. **a**, Schematic representation of the procedure used to extract cell-type-specific genomic DNA and nuclear RNA from cell nuclei labeled with cell-type-specific probes. Created with BioRender.com. **b**, Representative FANS plots showing the labeling of striatal cell nuclei with PrimeFlow probes specific for dopamine receptor D1-expressing (DRD1^+^) medium spiny projection neurons of the direct pathway (dMSNs), dopamine receptor D2-expressing (DRD2^+^) medium spiny projection neurons of the indirect pathway (iMSNs), somatostatin-expressing interneurons (SST^+^ INs, SST^+^ nuclei), fast-spiking interneurons expressing parvalbumin (PVALB^+^ INs, ETV1^+^ TAC3^−^ nuclei), primate-specific tachykinin precursor 3-expressing interneurons (TAC3^+^ INs, ETV1^+^ TAC3^+^ nuclei) and cholinergic interneurons expressing choline acetyltransferase (CHAT^+^ INs, TRPC3^+^ COL6A6^+^ nuclei). The probe against *PPP1R1B* labels all MSN nuclei. The detailed strategy used for sorting is described in Methods. **c**,**d**, Representative distribution of human FANS-seq (c) and ATAC-seq (d) reads mapped to genes expressed specifically in each of the striatal cell types studied. In panel d, arrows mark the position of annotated transcriptional start sites. The data are from a 41-year-old male control donor.

**Fig. 2:**
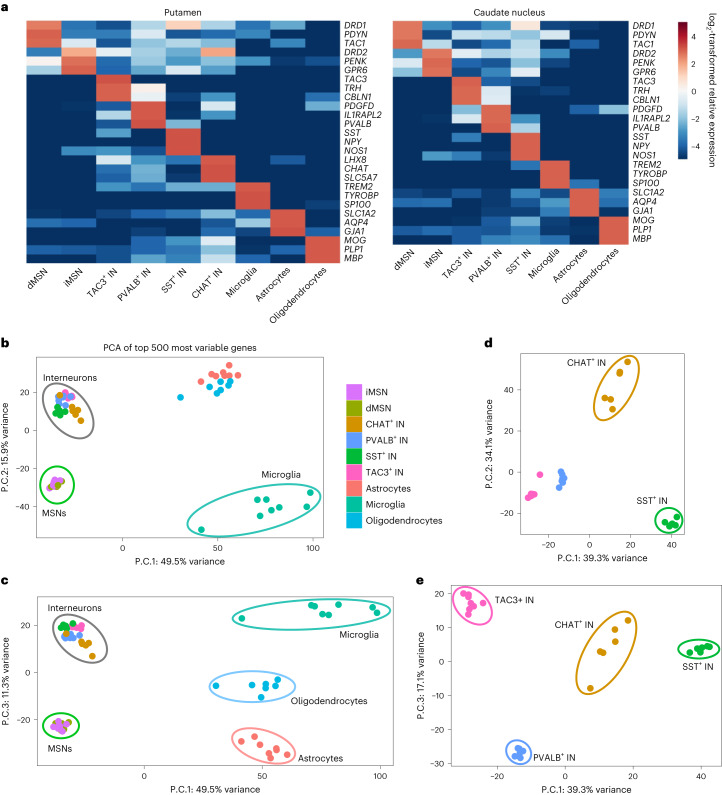
Purity and reproducibility of the isolation of striatal cell-type nuclei across the two striatal brain regions studied. **a**, Heatmaps depict log_2_-transformed relative expression level of cell-type-specific marker genes in each cell type, calculated based on the mean of DESeq2-normalized counts from six to eight control donors. **b**–**e**, Principal-component analysis (PCA) of control donor (*n* = 6–8 individuals) FANS-seq datasets from all putamen cell types (b,c) indicated that the first three principal components (P.C.) separated neuronal datasets from glial ones (P.C. 1), MSN datasets from those of interneurons (P.C. 2) and datasets of different glial cell types from each other (P.C. 3). For FANS-seq datasets from putamen interneurons (d,e), the major principal components separated the datasets according to interneuron subtype.

**Extended Data Fig. 1:**
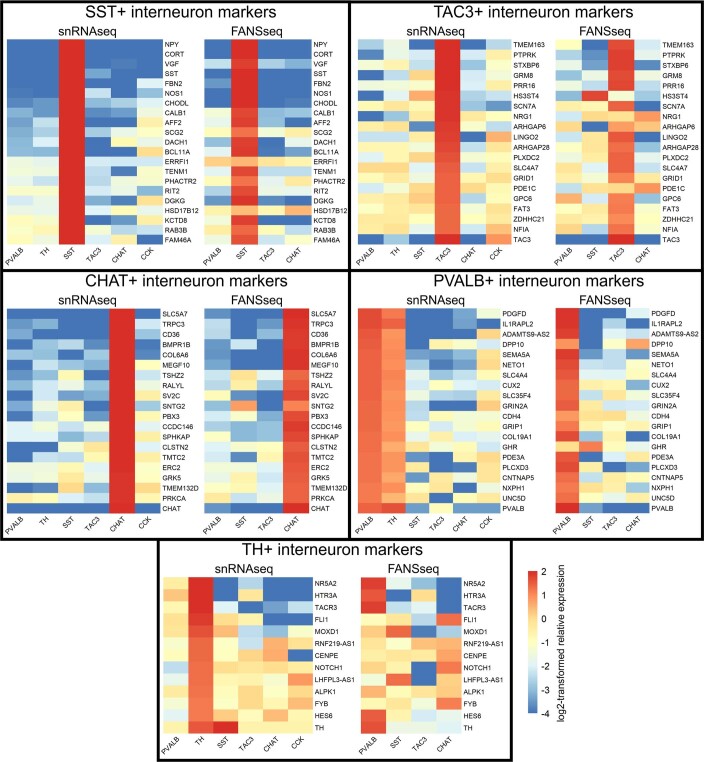
Comparison of interneuron populations collected using FANS to cell types defined from single-nucleus RNA sequencing of human striatum. Relative expression in each cell type was calculated based on DESeq2-normalized counts from 6-8 control donors, and was log_2_-transformed for visualization (see Methods). The marker genes specific for each interneuron subtype were selected based on single-nucleus RNA sequencing (snRNA-seq) data^19^. The FANS-isolated ETV1 + TAC3- population of Parvalbumin-expressing interneuron nuclei most likely captured both the major PVALB+ interneuron population and the related smaller PVALB + TH+ interneuron population defined as a separate subtype in snRNA-seq analysis^19^.

**Extended Data Fig. 2:**
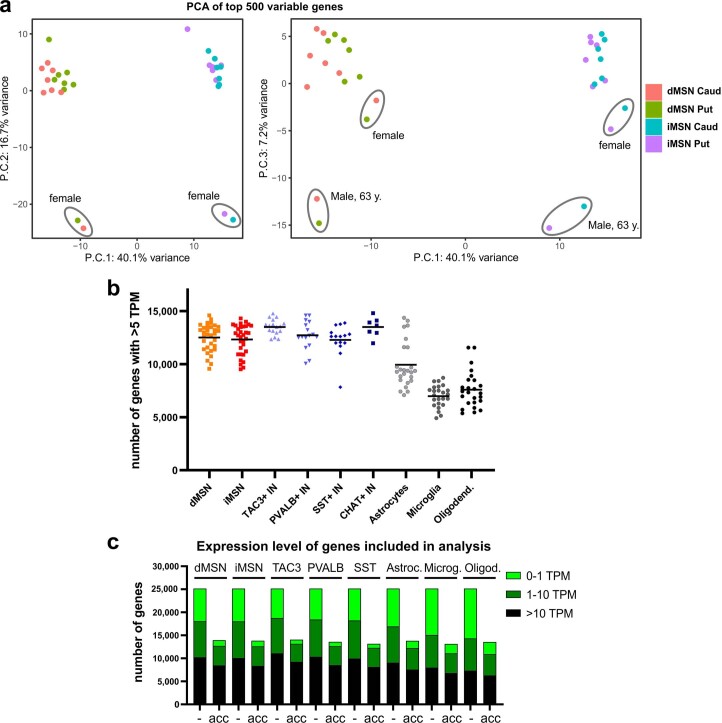
Characterization of FANS-seq datasets from control donors. **a**, Principal-component analysis of FANS-seq data from MSNs of caudate nucleus and putamen from 8 control donors. The main principal components separated datasets according to MSN subtype (P.C.1) and donor sex (P.C.2). Notably, none of the top principal components related to the brain region of origin (that is caudate nucleus or putamen). **b**, Number of genes with expression level above an arbitrary cutoff value of 5 transcripts per million (TPM) in individual FANS-seq datasets from control donors (mean is plotted). **c**, The number of genes with accessible transcriptional start sites (acc.), defined as having a consensus ATAC-seq peak at the transcriptional start sites (TSS), in striatal cell types from control donors. ATAC-seq datasets could not be produced from CHAT+ INs due to their low abundance in striatal tissue. Genes have been grouped according to average expression level in FANS-seq datasets from the specified cell type in control donors (*n* = 6-8 individuals). Excluding genes with inaccessible promoters removed a large proportion of genes with very low expression levels (<1 TPM) while affecting fewer genes with moderate to high expression (>10 TPM). See also Supplementary Note 1.

To characterize the expression profile, somatic mutations and chromatin accessibility of human striatal cell types, we further developed the FANS method^17^ (Fig. 1a). Nuclei were purified from samples dissected from human post-mortem caudate nucleus and putamen, stained with either antibody or RNA-binding probes specific for the nuclei of cell types of interest and resolved by passage through a fluorescence-activated cell sorter (Fig. 1a,b and Supplementary Table 1). The specificity of labeling probes and purity of isolated populations of nuclei were verified by generating RNA-sequencing (RNA-seq) libraries from their nuclear transcriptomes (FANS-seq) and comparing their gene expression profile to well-known cell-type markers and previously published data^18,19^ (Figs. 1c, d and 2, Extended Data Fig. 1 and Supplementary Table 2). This approach was used to generate comprehensive high-quality FANS-seq and ATAC-seq datasets for each neural cell type present in the human caudate nucleus and putamen (Extended Data Fig. 2 and Supplementary Note 1).

### *mHTT* CAG expands in vulnerable MSNs and resilient CHAT^+^ INs

**Extended Data Fig. 3:**
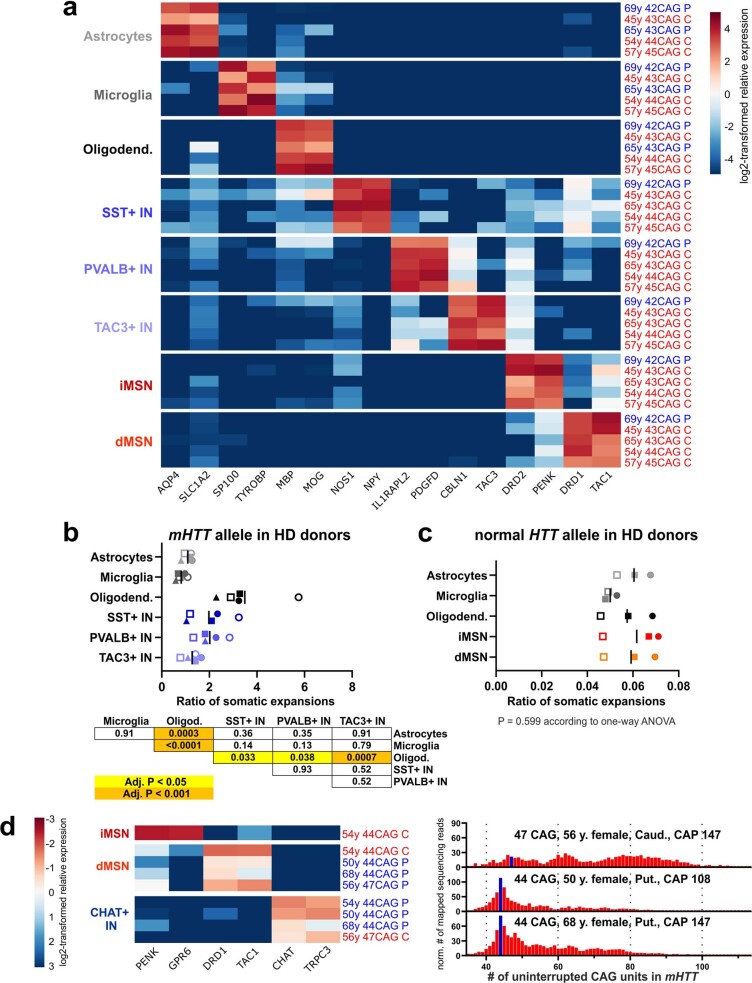
Further characterization of samples used for *mHTT* CAG repeat tract stability analysis. **a**, Relative expression level of marker genes of striatal cell types in the nuclear transcriptome of nuclei collected for *mHTT* CAG repeat tract analysis. Heatmap depicts log_2_-transformed relative expression in each sample (calculated based on DESeq2-normalized counts). The striatal region of origin is indicated by colored letters (blue P, putamen; red C, caudate nucleus). **b**, Comparison of the calculated ratio of somatic expansions of *mHTT* CAG tract in striatal cell types other than MSNs (*n* = 5 individual donors). The table presents adjusted *P*-values as calculated by Holm–Sidak’s multiple comparisons test post one-way ANOVA (*P* < 0.0001). See also Supplementary Note 2. **c**, Ratio of somatic expansions of normal *HTT* allele CAG tract in striatal cell types isolated from *n* = 3 HD donors with 21-25 uninterrupted repeats in normal HTT allele. *P* = 0.599 according to one-way ANOVA. A different symbol is used for each donor. **d**, (Left) Relative expression level of MSN and CHAT + IN marker genes in the nuclear transcriptome of nuclei collected for *mHTT* CAG repeat tract analysis (for description see **a**). (Right) Length distribution of *mHTT* CAG tract in CHAT + IN samples. Blue bar marks sequencing reads derived from the initial unexpanded CAG tract. y axes denote normalized number of sequencing reads mapped to reference sequences with different CAG tract lengths (normalized by scaling to 1,000 reads). Reads derived from the normal *HTT* allele are not shown.

Large somatic expansions of the *mHTT* exon 1 CAG tract in the striatum and cerebral cortex have been demonstrated by small-pool PCR^12^, and laser capture studies have suggested that somatic expansion can occur in both neurons and glia, albeit more frequently in neurons^15^. To address the specificity of somatic expansion of *mHTT* CAG tract quantitatively, and to understand whether it is correlated with cell loss in HD, we isolated nuclei of each striatal cell type by FANS (Supplementary Fig. 1), verified the purity of isolated populations of nuclei by analysis of marker gene expression in the nuclear transcriptomes (Extended Data Fig. 3a) and measured the length of *HTT* exon 1 CAG tract in genomic DNA isolated from these populations by Illumina-sequencing of amplicons derived from *HTT* exon 1^20^. Although the limited length of sequencing reads prevented the detection of CAG repeat lengths larger than 113 repeat units, reported to be present in a small minority of striatal cells^12^, the deep sequencing coverage over *HTT* CAG tract this method allows is expected to capture highly quantitative information from the vast majority of cells.

**Fig. 3:**
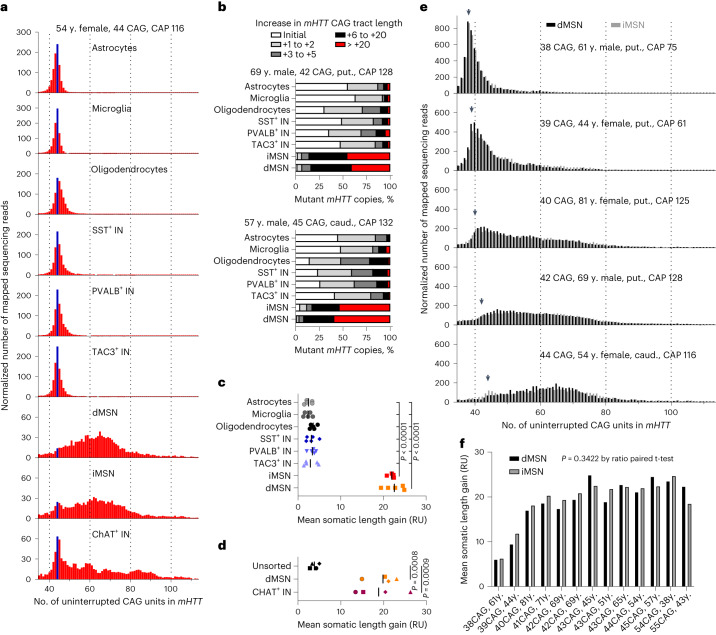
*mHTT* CAG tract undergoes somatic expansion in selected striatal neuron types. **a**, Length distribution of *mHTT* CAG tract in studied cell types of caudate nucleus and putamen of a 54-year-old female donor that carried a tract of 44 uninterrupted CAG units. Blue bar marks sequencing reads derived from the initial unexpanded CAG tract. *y* axes denote normalized number of sequencing reads mapped to reference sequences with different CAG tract lengths (normalized by scaling to 1,000 reads). Reads derived from the normal *HTT* allele are not shown. **b**, Frequency distribution of *mHTT* copies with different CAG tract length increases. Data are shown for striatal cell types of two donors that carried the most common HD-causing CAG tract lengths. **c**,**d**, Comparison of mean somatic length gain (measured in repeat units (RUs)). **c**, Although the mean somatic length gain of *mHTT* CAG tract was not different between dMSN and iMSN, comparison of each of these to any other striatal cell type showed a statistically significant difference (*n* = 5 individuals, *P* < 0.0001 by one-way analysis of variance (ANOVA), adjusted *P* < 0.0001 in Holm–Sidak’s multiple comparisons test). **d**, The mean somatic length gain of *mHTT* CAG tract was not different between dMSNs and CHAT+ interneurons, but comparison of each of these to unsorted nuclei showed a statistically significant difference (*n* = 4 individuals, *P* = 0.0004 by one-way ANOVA, adjusted *P* < 0.001 in Holm–Sidak’s multiple comparisons test). Different symbols are used for each of the four donors. **e**, Length distribution of *mHTT* CAG tract in MSNs of caudate nucleus and putamen in donors carrying *mHTT* alleles of reduced and full penetrance. Arrowhead indicates the initial unexpanded size of the CAG tract. **f**, Comparison of the mean somatic length gain of *mHTT* CAG tract. *n* = 13 individuals, *P* = 0.3422 between cell types, in ratio paired *t*-test (two sided). caud., caudate nucleus; put., putamen; y., year-old.

Analysis of genomic DNA of different cell types from five HD donors carrying most prevalent disease-causing CAG tract lengths (from 42 to 45 uninterrupted CAGs; see Extended Data Fig. 3a) revealed that *mHTT* CAG tract was relatively stable in glial cell types and SST^+^, TAC3^+^ and PVALB^+^ INs, having expanded by less than 5 repeat units in great majority of these cells (Fig. 3a–c, Supplementary Table 4, Supplementary Note 2 and Extended Data Fig. 3b). In contrast, only a small fraction of dMSNs and iMSNs had *mHTT* copies with the original inherited CAG tract length, and approximately half of these neurons had CAG tracts that were expanded by more than 20 repeat units (mean somatic length gain (MSLG) approximately 22 repeat units, Fig. 3a–c; Methods, ‘HTT and ATXN3 CAG tract sizing’).

Because the scarcity of tissue available prevented us from isolating striatal CHAT^+^ IN nuclei from all but one of these five initially characterized HD donors, we isolated nuclei of this very rare cell type from three additional HD donors. Analysis of these samples revealed that *mHTT* CAG tract undergoes large expansions also in CHAT^+^ INs (MSLG approximately 18 repeat units; Fig. 3a,d, Extended Data Fig. 3d and Supplementary Table 4).

Pairwise comparisons of the two MSN types from donors carrying a wider range of initial repeat lengths revealed that the extent of repeat expansion was dependent on initial repeat length. Although the comparison is confounded by differences in age at death, more modest expansion was observed for *mHTT* alleles with reduced penetrance (CAG tract lengths <40 repeats) relative to longer, fully penetrant alleles (Fig. 3e,f). Interestingly, although iMSNs have been reported to be more vulnerable of the two MSN subtypes^21^, there was no significant difference in MSLG between dMSNs and iMSNs (Fig. 3f; *P* = 0.3422 by ratio paired *t*-test).

Taken together, these data support the hypothesis that extensive somatic expansion of the *mHTT* CAG tract is required for the vulnerability of MSNs in HD. However, given previous studies demonstrating that CHAT^+^ INs in the striatum are not lost in HD^3,4^, our results suggest that expansion of the *mHTT* CAG tract is not sufficient to cause neuronal loss in HD.

### Instability of the *mHTT* CAG tract in the HD cerebellum

**Fig. 4:**
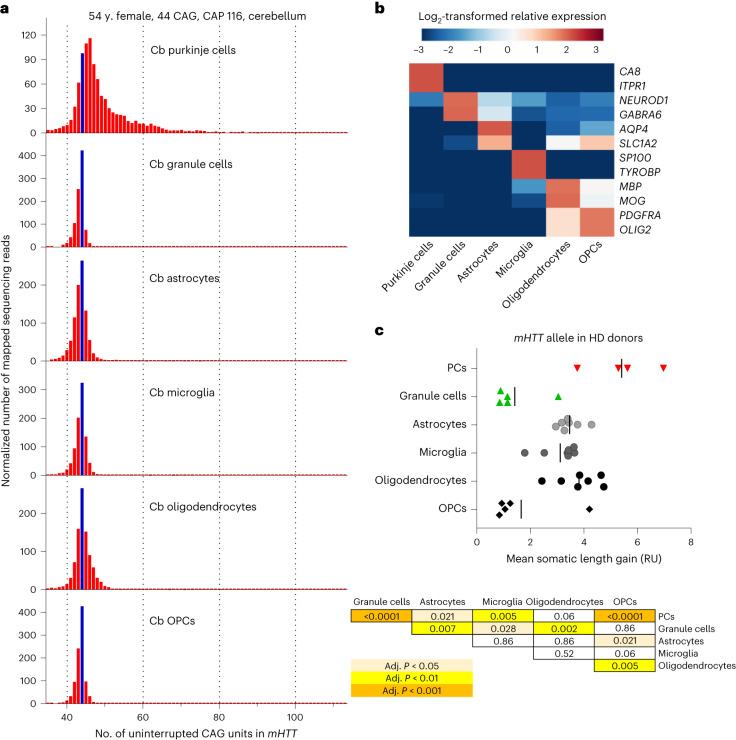
Expansion of *mHTT* CAG tract in cerebellar PCs. **a**,**b**, Length distribution of *mHTT* CAG tract (a) and cell-type marker gene expression (b) in cerebellar (Cb) cell nuclei isolated from a 54-year-old female donor that carried a tract of 44 uninterrupted CAG units. Blue bar marks sequencing reads derived from the initial, unexpanded CAG tract. Reads derived from the shorter normal *HTT* allele are not shown. **b**, Heatmap depicts log_2_-transformed relative expression in each sample (calculated based on DESeq2-normalized counts). **c**, Comparison of the mean somatic length gain of *mHTT* CAG tract in cerebellar cell types from four to seven HD donors (*n* = 4 individuals for PCs, *n* = 5 for granule cells, *n* = 7 for astrocytes, microglia and oligodendrocytes, and *n* = 5 for oligodendrocyte progenitor cells (OPCs)). The table presents adjusted *P* values as calculated by Holm–Sidak’s multiple comparisons test following one-way ANOVA (*P* < 0.0001). The variability in somatic CAG expansion observed for PC samples from different donors can most likely be attributed to the rarity of this cell type (<0.01% of all nuclei), which made it extremely difficult to collect samples entirely free of contamination with nuclei of ‘non-expanding’ cell types. Alternatively, the extent of somatic CAG expansion in PCs could be variable in the donors we analyzed, given the reported variability of PC loss between HD patients^24^.

The loss of cerebellar Purkinje cells (PCs) in several spinocerebellar ataxias (SCA1, SCA2, SCA6 and SCA7) where the causal elongated CAG tracts undergo germline expansion has suggested that, in these disorders, somatic CAG expansion may occur in PCs, but this has not been documented in the cerebellum of HD donors^22^. Although the viability of PCs in HD has been a matter of debate, ataxia is not an uncommon symptom in HD patients^23^, and recent stereological studies have demonstrated that PC loss occurs in the cerebellum in HD cases with predominant motor symptoms^24^. Given these data, we measured *mHTT* CAG instability in cerebellar cell types in several HD donors (Supplementary Table 1). Although the degree of *mHTT* CAG expansion in PCs (MSLG approximately 5 repeat units) was relatively modest compared to MSNs, the tract had expanded more in PCs than in other cerebellar cell types except oligodendrocytes (Fig. 4a–c and Supplementary Table 4; *P* < 0.0001 by one-way ANOVA, adjusted *P* < 0.05 in Holm–Šidak’s multiple comparisons test in comparisons of PCs to other cell types except oligodendrocytes [*P* = 0.0617]). The instability of *mHTT* CAG repeat in PCs relative to cerebellar granule cells is similar to that seen for the mutant *ATN1* CAG repeat causing dentatorubral pallidoluysian atrophy^25,26^. Our data indicate that both in striatum and cerebellum the *mHTT* CAG tract is somatically unstable in selected neuron types and much more stable in other neuron types and glial cells.

### Striatal MSNs are prone to somatic CAG expansion

Preferential somatic expansion of the *mHTT* CAG tract in selected striatal neuron types could be due to cell-type-specific properties of the *HTT* locus (that is, MSN and CHAT^+^ IN-specific factors acting in *cis*), putative cell-type-specific factors acting in *trans* (for example DNA repair proteins) or a combination of the two. Because the expansion-promoting effect of putative *trans*-factors would not necessarily be limited to the *mHTT* locus, we asked whether MSNs have a propensity to expand long and pure CAG or CTG tracts at other genomic loci as well. Because transcription through the repeat seems to be a prerequisite for somatic expansions^27^, we chose to analyze the CAG repeat in *ATXN3* gene because, as is the case for *HTT*, its transcription is relatively uniform across striatal cell types (Supplementary Fig. 2a).

**Fig. 5:**
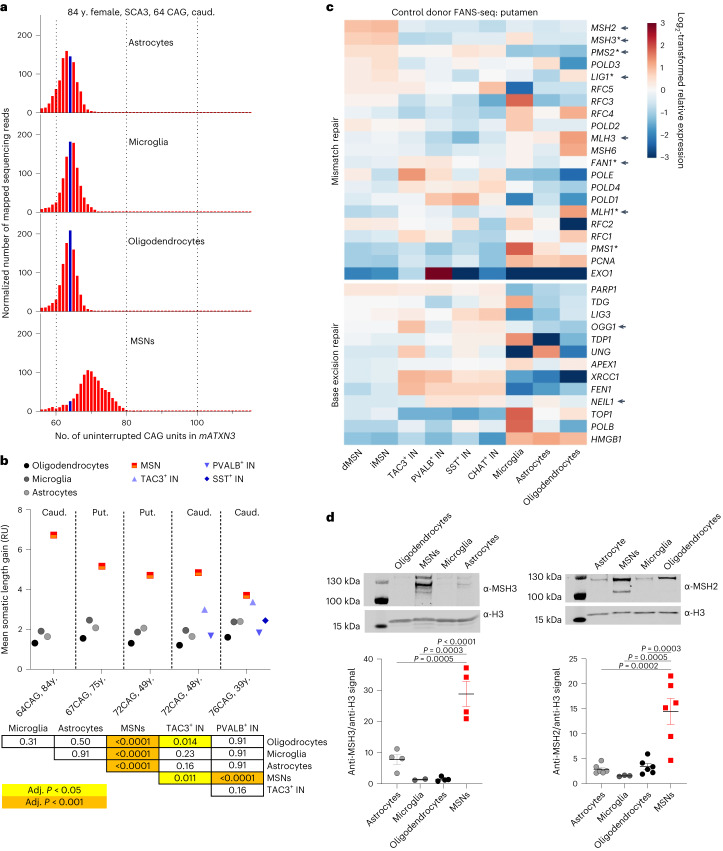
MSNs are prone to somatic expansion of *mATXN3* CAG tract and have elevated expression of nuclear MSH2 and MSH3 proteins. **a**, Length distribution of *mATXN3* CAG tract in cell types of caudate nucleus of an 84-year-old female donor that carried a tract of 64 uninterrupted CAG units. Size of the initial unexpanded CAG tract is marked with a blue bar. Reads derived from the normal *ATXN3* allele are not shown. **b**, Comparison of the mean somatic length gain of *mATXN3* CAG tract in striatal cell types from SCA3 donors (*n* = 5 individuals for MSNs, astrocytes, microglia and oligodendrocytes, *n* = 2 TAC3^+^ INs and PVALB^+^ INs, and *n* = 1 for SST^+^ INs). The table presents adjusted *P* values as calculated by Holm–Sidak’s multiple comparisons test following one-way ANOVA (*P* < 0.0001). **c**, Heatmaps depict log_2_-transformed relative expression of MMR and BER genes in cell types of putamen, calculated based on DESeq2-normalized counts from six to eight control donors. Genes identified as HD age at onset-modifying candidates or known to influence CAG tract instability in HD mouse models are marked with an asterisk or arrowhead, respectively. **d**, Representative immunoblots and quantification of anti-MSH3/anti-H3 (left) and anti-MSH2/anti-H3 signal ratio (right) in unfixed nuclei isolated from the putamen of control donors. These ratios were higher for MSNs compared to other cell types analyzed (*P* < 0.0001 by one-way ANOVA, adjusted *P* ≤ 0.0005 in Tukey’s multiple comparisons test). For anti-MSH3, *n* = 4 individuals for MSNs, astrocytes, and oligodendrocytes, and *n* = 2 individuals for microglia. For anti-MSH2, *n* = 6 individuals for MSNs, astrocytes and oligodendrocytes and *n* = 3 individuals for microglia. Data are presented as mean ± standard error of the mean (s.e.m.). Full-length blots are provided as Source Data. Source data

We isolated glial cell and MSN nuclei from striatal tissue of five donors with spinocerebellar ataxia 3 (SCA3) and striatal interneuron nuclei from two SCA3 donors, all carrying a long CAG repeat in the mutant *ATXN3* allele (*mATXN3*) (Supplementary Table 1). Although there were no clear signs of MSN loss even in the oldest SCA3 donors analyzed, as judged by the abundance of large NeuN^+^ nuclei in striatal homogenates (Supplementary Fig. 2b), the m*ATXN3* CAG tract was clearly more unstable in the MSNs relative to glial cells and interneurons (Fig. 5a,b and Supplementary Table 4; MSLG approximately 5 repeat units in MSNs). These data indicate that MSNs have a propensity to expand long CAG tracts at other genomic loci and support the hypothesis that *mHTT* exon 1 CAG tract instability is modulated by rate-limiting *trans*-acting factors expressed at different levels in striatal cell types.

**Extended Data Fig. 4:**
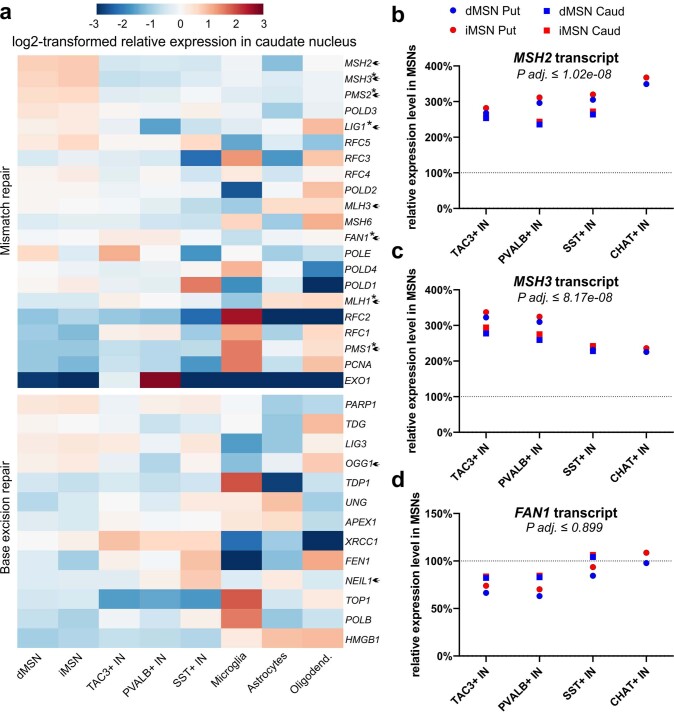
Relative expression level of MMR and BER genes in cell types of caudate nucleus. **a**, Heatmaps depict log_2_-transformed relative expression in each cell type, calculated based on the mean of DESeq2-normalized counts from 7-8 control donors. Genes identified as HD age at onset-modifying candidates are marked with an asterisk. Genes known to influence CAG tract instability in HD mouse models are marked with an arrowhead. **b-d**, Relative levels of *MSH2* (**b**), *MSH3* (**c**) and *FAN1* (**d**) transcripts in MSNs compared to interneurons, calculated with DESeq2 using cell type-specific FANS-seq data from control donors. The largest *P* adj. value of all MSN vs. interneuron comparisons (by DESeq2, *P* adjusted for multiple comparisons) is indicated for each gene. For caudate nucleus samples: *n* = 8 individuals for dMSNs, iMSNs, TAC3 + IN, PVALB + IN and SST + IN. For putamen samples: *n* = 8 individuals for dMSNs, iMSNs, TAC3 + IN, *n* = 7 individuals for PVALB + IN and *n* = 6 individuals for SST + IN.

To identify these *trans*-acting factors that may explain preferential CAG expansion in MSNs, we compared the FANS-seq expression profiles of striatal cell types in control donors. We focused on genes coding for DNA MMR and base-excision repair (BER) proteins, as several of these proteins have been shown to affect repeat instability in model systems^28^, and because several MMR genes are represented among candidate genes identified as age of motor symptom onset modifiers in HD mutation carriers^14,29^. We found that transcript levels of *MSH2* and *MSH3*, encoding MMR proteins that form the MutSβ complex, were more than twofold higher in both dMSNs and iMSNs compared to other striatal neurons, including CHAT^+^ INs, and this difference was consistent across neuron types in both putamen and caudate nucleus (Fig. 5c, Extended Data Fig. 4a–c and Supplementary Note 3).

To determine whether the FANS-seq data accurately reflected nuclear protein levels for these factors in abundant striatal cell types, we measured MSH2 and MSH3 levels by western blotting of nuclear lysates from MSNs, microglia, astrocytes and oligodendrocytes (Fig. 5d). The ratio of both MSH2 and MSH3 to chromatin, assessed by anti-H3 signal, was significantly higher in MSN nuclei compared to glial cells (Fig. 5d; *P* < 0.0001 by one-way ANOVA, adjusted *P* ≤ 0.0005 in Tukey’s multiple comparisons test in comparisons involving MSNs). It is well established that the level of *Msh2* and *Msh3* modulates the extent of somatic CAG expansions seen in the striatum of HD mouse models carrying expanded CAG tracts^30–34^. Therefore, our observations suggest that elevated levels of the two components of MutSβ may explain the enhanced CAG expansions in human MSNs.

### MutSβ suppresses FAN1’s excision of excess slipped-CAG DNA

**Fig. 6:**
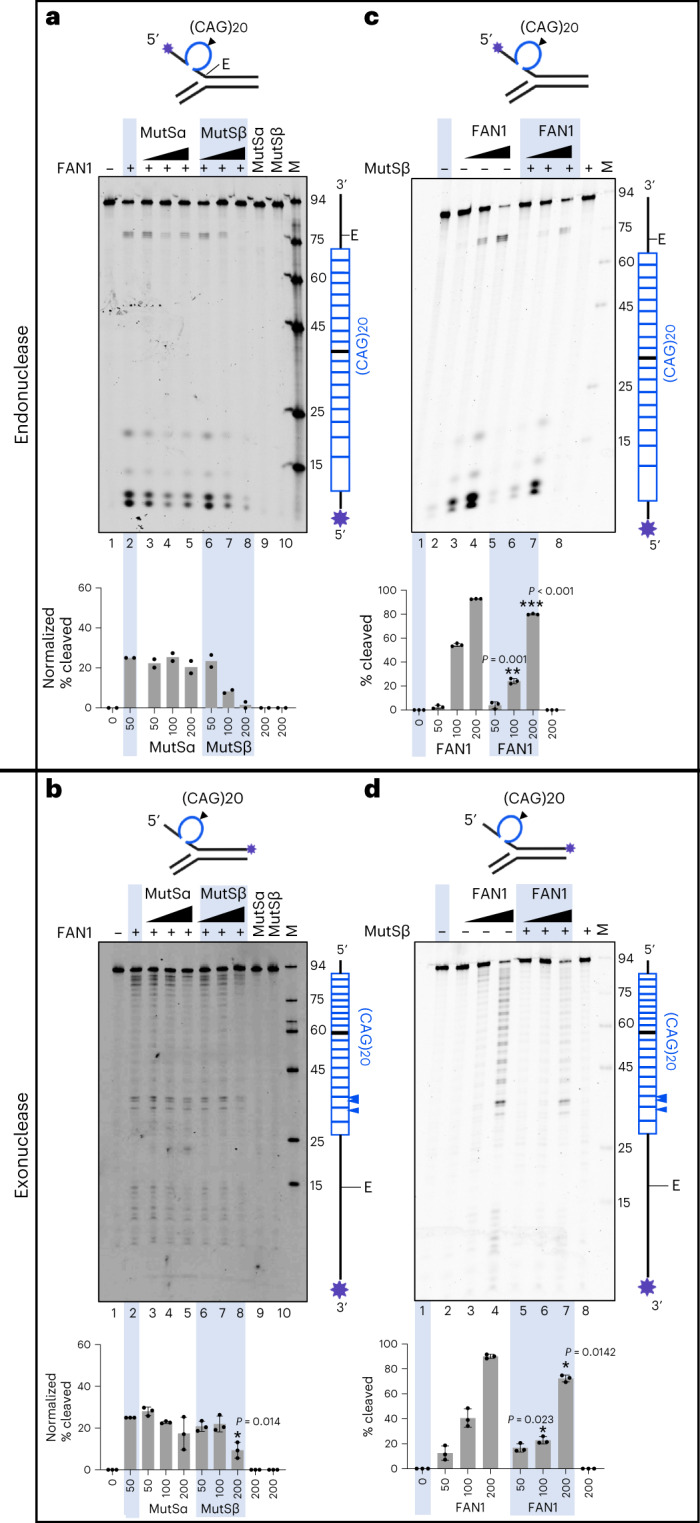
MutSβ and FAN1 levels competitively affect FAN1’s nuclease excision of excess slipped-CAG DNAs. (CAG)20-slip-out DNA substrates (schematics) mimic putative intermediates of expansion mutations^40^. Endo- and exonucleolytic activities can be distinguished by fluorescein amidite (FAM)-labeling at 5′ or 3′ ends of the (CAG)20 strand, respectively (indicated by an asterisk); in this manner, only the labeled strand and its digestion products are tracked. ‘E’ is elbow at the dsDNA-ssDNA junction. **a**,**b**, MutSβ not MutSα inhibits FAN1. Protein-free undigested slip-out DNA substrate (100 nM), lane 1. Slip-outs were preincubated with buffer, lane 2, or increasing concentrations of purified human MutSα (50, 100 or 200 nM), lanes 3–5; or MutSβ (50, 100 or 200 nM), lanes 6–8. Nuclease digestions were initiated by addition purified human FAN1 (50 nM). Lanes 9 and 10 have slip-out DNA and only MutSα (50 nM) or only MutSβ (50 nM), ensuring these purified proteins are nuclease-free. **c**,**d**, Increasing FAN1 concentration can overcome MutSβ-mediated inhibition of cleavage. Protein-free undigested slip-out (100 nM), lane 1. Slip-outs were preincubated with buffer, lanes 1–4 or with MutSβ (200 nM), lanes 5–7. Nuclease digestions were initiated by adding increasing amounts of FAN1 (50, 100 or 200 nM), lanes 2–4 and 5–7. Lane 8 has slip-out and only MutSβ (50 nM). For gels in panels a and b, the percentage cleavage for each reaction was normalized to cleavage levels with FAN1 alone (lane 2), and these levels were graphed (GraphPad prism 9.1). For gels in panels c and d, the percentage cleavage for each reaction were graphed. The vertical schematic to the right of each gel indicates the location of cleavage sites along the FAM-labeled DNA strand. ‘E’ is elbow at the dsDNA-ssDNA junction, blue arrowheads represent cleavage hotspots. Results of two-sided unpaired *t*-test are indicated (versus FAN1 alone in panel b, or versus ‘no FAN1’ in panels c and d). *n* = 2 experiments for panel a, *n* = 3 experiments for the other panels and mean ± standard deviation (s.d.) are plotted.

**Extended Data Fig. 5:**
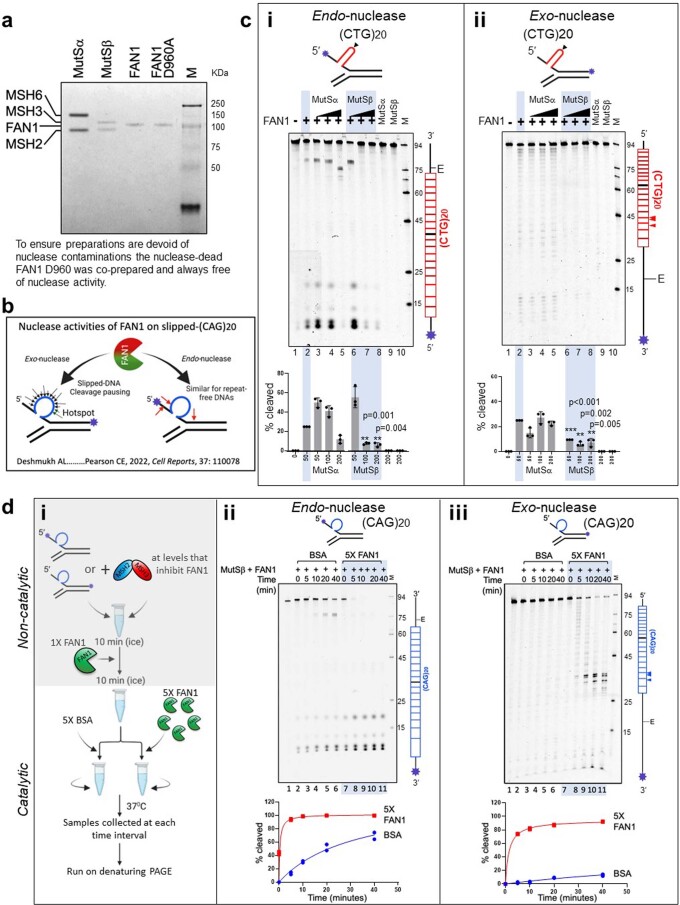
MSH2, MSH3, MSH6, and FAN1 proteins, nuclease assay, (CTG)20 digestion. **a**, A representative Coomassie brilliant blue-stained SDS-PAGE of purified human MutSα (MSH2-MSH6), MutSβ (MSH2-MSH3), FAN1 and nuclease-dead FAN1^p.D960A^ (purified in parallel to ensure an absence of contaminating nuclease activity in preparations), expressed in Sf9 cells using baculoviral overexpression. Both MutSα and MutSβ preparations were free of nuclease contamination (see **c** panels i and ii, lanes 9 and 10). Purity and activity were assessed for ~10 preparations, with consistent results. **b**, Schematic of FAN1 *exo*-nucleolytic and *endo*-nucleolytic cleavage sites on slipped-(CAG)20 DNA substrates labeled at the 3’ or 5’ ends, respectively. FAN1’s *exo*-nucleolytic ‘nibbling’ of excess repeats parallels the ‘inchworm’ expansions in patient brains, suggesting a role for FAN1 in regulating repeat instability^46^. *Endo*- and *exo*-nucleolytic activities can be distinguished by fluorescein amidite (FAM)-labeling at 5’ or 3’ ends of the (CAG)20 strand, respectively (asterisk); in this manner, only the labeled strand and its digestion products are tracked. **c**, Slipped-(CTG)20 DNAs are digested by *endo*- and *exo*-nucleolytic activities of FAN1, which can be inhibited by MutSβ, but not by MutSα. FAM-labelled CTG slip-out oligonucleotides (schematic, arrowhead indicated center of repeat tract) mimic putative intermediates of expansion mutations. *Exo*- and *endo*-nuclease activities were determined by labeling either 3′- or 5′-end of (CTG)20 strand, respectively (asterisk). ‘E’ is elbow at the dsDNA-ssDNA junction. Slip-out DNA substrates (100 nM) were preincubated with increasing amounts of purified human MutSα or MutSβ (0-200 nM), and nuclease digestion was initiated by addition purified human FAN1 (50 nM). Vertical schematic to the right of each gel indicates location of cleavage sites along the FAM-labeled DNA strand. Percentage cleavage (densitometry) for each reaction was then normalized to cleavage levels for FAN1 alone, and these levels were graphed (*n* = 3 experiments, mean ± s.d. plotted). Results of two-sided unpaired *t*-test are indicated (vs. ‘FAN1 alone’ in panels i and ii). **d**, Excess FAN1 overcomes MutSβ-mediated inhibition. (**i**) Flowchart outlining the competition experiment. Human MutSβ (200 nM), FAN1 (50 nM) and (**ii**) 5’- or (**iii**) 3′-FAM-labeled CAG slip-outs (100 nM), that is conditions established to be completely inhibited by MutSβ, were preincubated in non-catalytic conditions (on ice). Reactions were split at the 10-min time point and challenged with 5× BSA, to control for macromolecular crowding (lanes 2–6) or an excess (5×) of FAN1 (lanes 7–11). Aliquots were taken for analysis at different timepoints (0, 5, 10, 20 and 40 minutes) and cleavage products were quantified (*n* = 2 experiments).

*FAN1* was identified as a modifier of HD disease^13^, and the nuclease activity of FAN1 has been shown to suppress CAG expansions in the central nervous system of HD mice and in cells derived from HD patients^35–40^. Unlike the levels of *MSH2* and *MSH3* transcripts, *FAN1* transcript levels are not higher in MSNs compared to other striatal neuron types (Extended Data Fig. 4d). We asked whether a higher MutSβ to FAN1 ratio, as predicted based on elevated *MSH2* and *MSH3* expression in MSNs, might affect the excision rates of putative expansion intermediate DNA structure by the FAN1 nuclease. To answer this, we used purified recombinant human proteins (Extended Data Fig. 5a) and slipped-(CAG)20 DNA substrates, previously demonstrated to be cleaved by both endo- and 5′→3′ exonucleolytic activities of FAN1 (detailed in Extended Data Fig. 5b)^40^. Addition of increasing concentrations of MutSβ lead to progressive and substantial inhibition of endo-nucleolytic excision by FAN1 (Fig. 6a, compare lane 2 with lanes 6–8). In contrast, addition of increasing concentrations of MutSα, a dimer of MSH2 and MSH6, did not inhibit cleavage significantly (Fig. 6a, compare lane 2 with lanes 3–5). Slipped-(CTG)20 DNA substrate could also be excised by FAN1, and this was inhibited significantly by MutSβ, but not MutSα (Extended Data Fig. 5c panel i). Next, we tested the effect of the MutS complexes on FAN1s exo-nucleolytic digestion of slip-out DNA substrates, where ‘nibbling-like’ cleavage occurred throughout the repeat tract (Fig. 6b and Extended Data Fig. 5c panel ii, compare lane 1 with lane 2). *Exo*-nucleolytic cleavage of both slipped-CAG(20) and slipped-(CTG)20 was inhibited by MutSβ, but not MutSα (Fig. 6b and Extended Data Fig. 5c panel ii, compare lane 2 with lanes 6–8 and lanes 3–5). Thus, unlike MutSα, MutSβ inhibits FAN1’s exo- and endonucleolytic excision of excess CAG and CTG slip-outs. The inhibition of FAN1 excision by MutSβ was also significant for slip-outs in the context of anchored duplex flanks, and this effect was evident for varying slip-out sizes, with 2, 4, 8 and 14 excess repeats (Supplementary Fig. 4).

Addition of increasing concentrations of FAN1 led to increased endo- and exonucleolytic digestion of slip-out DNA even in the presence of MutSβ (Fig. 6c,d, compare lane 5 with lanes 6 and 7), and excess FAN1 could also overcome inhibition of slip-out DNA cleavage by pre-bound MutSβ in a competition experiment (Extended Data Fig. 5d, compare lanes 7–11 with lanes 2–6, in both panels ii and iii). As FAN1 does not interact with MutSβ^38^, our results support a model where CAG and CTG slip-out DNA excision rates are determined by competitive binding to either MutSβ or FAN1, thereby offering an explanation to how differences in the relative level of MutSβ to FAN1 could result in CAG expansion or stabilization in different cell types.

### Altered gene expression in HD progression

**Fig. 7:**
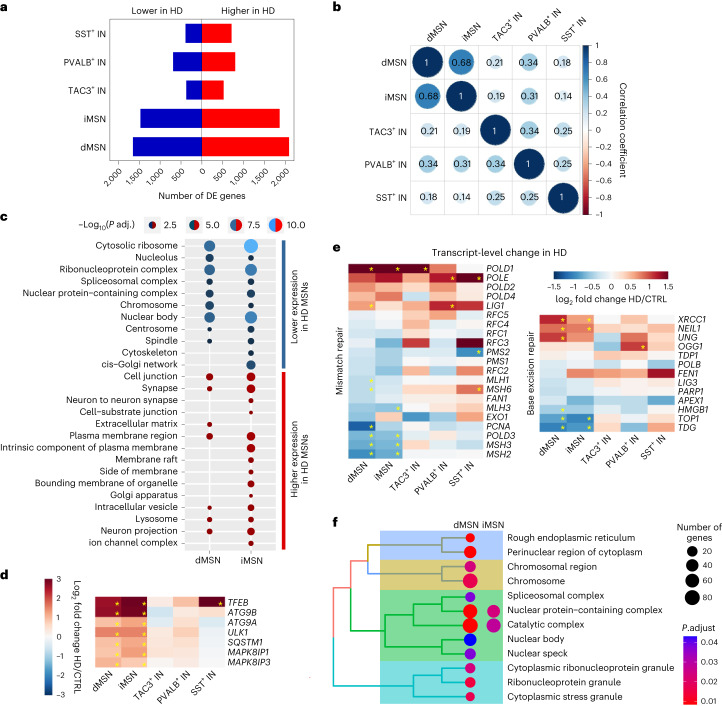
Disease-associated gene expression changes in striatal neuron types. **a**, Number of differentially expressed (DE) genes (*P*_adj_ < 0.05 by DESeq2, adjusted for multiple comparisons) in the comparison of HD (*n* = 7 individuals for dMSNs and iMSNs, *n* = 6 individuals for all interneuron types) and control donor (*n* = 8 individuals) FANS-seq datasets from putamen or caudate nucleus. **b**, Correlation analysis of disease-associated expression changes of genes expressed in all striatal neuronal types studied. **c**, Selected nonredundant GOCC terms from enrichment analysis of genes with disease-associated expression changes in iMSN or dMSN (*P*_adj_ < 0.05 by DESeq2), but not in any interneuron type (*P*_adj_ > 0.05 in all HD versus control interneuron comparisons). **d**,**e**, Heatmaps depicting disease-associated changes in transcript levels of selected genes regulating autophagosome formation and transport (d), and transcript levels of MMR and BER genes (e). Statistically significant differences are marked with an asterisk (*P*_adj_ < 0.05 by DESeq2, adjusted for multiple comparisons). CTRL, control. **f**, GOCC terms enriched for genes that were identified as essential for MSN viability in wild-type mice^43^ and are also downregulated in HD dMSNs (440 genes) or iMSNs (365 genes). GOCC terms enriched with less than 10 downregulated genes are omitted from the plot. For panels c and f, the significance threshold for enri**c**hment analysis was: *q* value < 0.05, *P*_adj_ < 0.05, adjusted for multiple comparisons after hypergeometric test with clusterProfiler.

**Extended Data Fig. 6:**
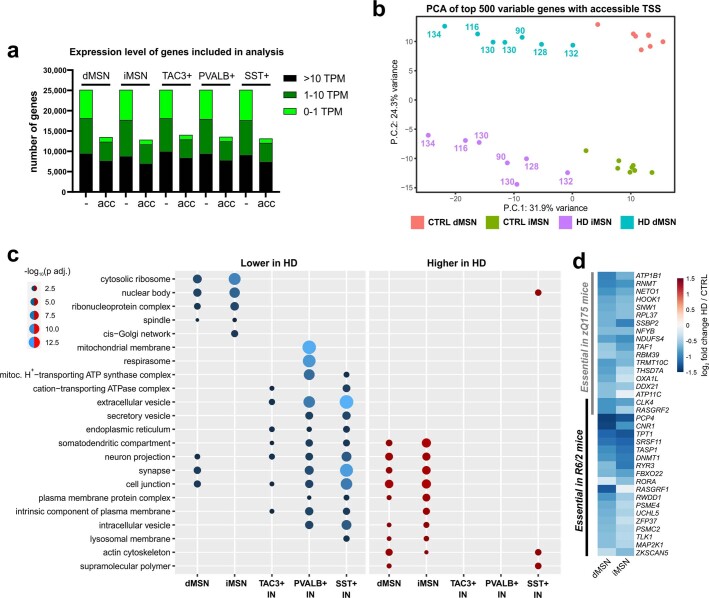
Further characterization of HD-associated gene expression changes in striatal neurons. **a**, The number of genes with accessible transcriptional start sites (acc.), defined based on the overlap of TSS and consensus peaks in ATAC-seq data from HD and control donor MSNs, and control donor interneurons. Genes have been grouped according to their average expression level in FANS-seq datasets from HD donors (TPM, transcripts per million). More than 98% of genes with accessible promoters in HD MSNs had accessible promoters also in MSNs from control donors. Due to the low abundance of striatal interneurons and limited amounts of striatal tissue available from HD donors, ATAC-seq datasets could not be produced from the interneurons of HD donors. Therefore, striatal interneuron FANS-seq datasets were filtered to include only those genes that had accessible promoters in control donors. **b**, Principal-component analysis of FANS-seq data from putamen and caudate nucleus MSNs from HD and control donors, performed after the exclusion of genes with inaccessible TSSs. The calculated CAP100 scores are shown for each HD donor. Note that disease status (P.C.1) and MSN subtype (P.C.2) were the main sources of variance in these datasets. **c**, Selected nonredundant Gene Ontology Cellular Component terms from enrichment analysis of genes with disease-associated expression changes (*P*_adj_ < 0.05 by DESeq2 after adjusting for multiple comparisons) in each striatal neuron type HD (*n* = 7 individuals for HD dMSNs and HD iMSNs, *n* = 6 individuals for HD interneurons, *n* = 8 individuals for all cell types from control donors). Significance threshold for enrichment analysis: *q* value < 0.05, *P*_adj_ < 0.05, adjusted for multiple comparisons after hypergeometric test with clusterProfiler. **d**, Genes essential for MSN viability in the zQ175 and R6/2 mouse models of HD^43^ that are also downregulated in HD iMSNs or dMSNs by more than a third (log_2_FC < −0.6, *P*_adj_ < 0.01 by DESeq2, adjusted for multiple comparisons). See also Supplementary Note 5.

To gain further insight into the molecular events that may play a role in somatic expansion or contribute to *mHTT* toxicity, we sequenced the nuclear transcriptomes of striatal MSNs and TAC3^+^, SST^+^ and PVALB^+^ INs from the putamen or caudate nucleus of six or seven HD donors (Supplementary Tables 1 and 2). We limited the comparative analysis of HD and control donors’ (*n* = 8) FANS-seq data to genes that had accessible promoters in cell type of interest (as indicated by ATAC-seq), reasoning that this would allow us to minimize the number of possible false-positive differences in gene expression that might have resulted from contaminating ambient transcripts, FACS sorting impurities or contamination with genomic DNA (Supplementary Note 1 and Extended Data Fig. 6a). As expected, disease-associated changes (Fig. 7a and Supplementary Tables 5 and 6) were well correlated between dMSNs and iMSNs, but the correlation was poor in comparisons involving other neuron types (Fig. 7b and Extended Data Fig. 6b). This result shows that the majority of disease-associated transcript-level changes are not common to all striatal neuron types.

#### Gene ontology analysis

To identify cellular processes that are affected by disease-associated gene expression changes that take place only in MSNs, thereby correlating with the presence of more toxic mHTT species in these neurons, we analyzed which gene ontology cellular component (GOCC) terms were enriched for genes that were up- or downregulated in MSNs but did not display these changes in expression in any of the interneuron populations studied. The results indicated that many genes downregulated specifically in MSNs are involved in ribosomal biogenesis (GOCC terms ‘cytosolic ribosome’ and ‘nucleolus’), pre-mRNA maturation (GOCC terms ‘nuclear body’ and ‘spliceosomal complex’) and other nuclear functions (Fig. 7c). Although transcripts of mitochondrial oxidative phosphorylation pathway genes have been reported to be downregulated in HD MSNs^41^, we noticed that this disease-associated change is much more evident in the nuclear transcriptome of PVALB+ INs (Extended Data Fig. 6c and Supplementary Fig. 5a). The full lists of GOCC terms enriched for MSN-specific changes and overall changes in all neuron types can be found in Supplementary Tables 7 and 8, respectively.

The GOCC terms enriched for genes that have increased expression in MSNs in the HD donor data include many terms that indicate alterations in membrane protein function. ‘Neuron projection’, ‘Synapse’ and ‘Lysosome’ were among GOCC terms enriched for genes upregulated specifically in both MSN subtypes (Fig. 7c, Extended Data Fig. 6c and Supplementary Fig. 5b). We observed also that genes central to the regulation of lysosomal biogenesis and autophagy were among the top upregulated genes in MSNs. For example, the transcripts of transcription factor TFEB, which has been shown to be essential for regulation of many genes in these pathways^42^, are strongly elevated in HD MSNs (Fig. 7d). The observations that TFEB is essential for MSN survival in mice in the presence of *mHTT*^43^ and can lower striatal mHTT levels^44^ point to the relevance of its induction for MSN survival. Several genes encoding proteins essential for autophagy are also induced in HD, including *ATG9B*, *ATG9A*, the gene encoding HTT-interacting protein ULK1 involved in autophagosome formation^45^, *MAPK8IP1* and *MAPK8IP3*, which encode proteins involved in retrograde transport of autophagosomes^46^, and *SQSTM1*, the gene encoding HTT-interacting autophagy cargo receptor p62 (ref. ^45^) (Fig. 7d and Supplementary Note 4).

#### DNA repair pathways

Given the expansions of *mHTT* CAG tract in MSNs, we investigated whether there are disease-associated changes in the transcript levels of genes encoding MMR and BER proteins. Notably, we found that, in HD MSNs, *MSH2* and *MSH3* expression levels are significantly reduced relative to MSNs from control donors, whereas *POLD1*, coding for the large catalytic subunit of the DNA polymerase delta complex, undergoes a disease-associated upregulation that is not entirely specific to MSNs (Fig. 7e). Notably, *POLD1* was recently identified as a candidate modifier of HD^47^. Although further validation will be required to confirm these changes, our data point to clear distinctions in the regulation of DNA repair pathways in MSNs compared to interneurons.

#### Genes required for MSN viability and functionality

To predict which cellular functions would be affected negatively by HD-associated transcript-level changes, we identified genes downregulated in HD MSNs that have also been shown to be required for the viability of MSNs in mice^43^ (Supplementary Table 10). Analysis of GOCC terms enriched for these genes revealed that many of the HD-associated expression changes could be affecting MSN viability through their effect on nuclear functions and RNA metabolism (Fig. 7f and Supplementary Note 5). We also noted that the transcript levels of MSN-enriched *ANO3* and *PDE10A* undergo large disease-associated decreases equivalent in magnitude to complete silencing of these genes in >45% and >60% of the remaining MSNs, respectively (Supplementary Fig. 7, *ANO3* transcript log_2_ fold change −1.35 and −0.86 for dMSNs and iMSNs, *PDE10A* transcript log_2_ fold change −1.44 and −1.33 for dMSNs and iMSNs). As missense mutations in *ANO3* are known to cause dystonia (https://omim.org/entry/615034) and *PDE10A* mutations are known to cause childhood-onset hyperkinetic movement disorders (https://omim.org/entry/616921, in some cases with striatal degeneration: https://omim.org/entry/616922), it is likely that these transcript-level changes have a substantial effect on the function of a large fraction of remaining MSNs in HD.

## Discussion

Here, we have used FANS^17^ to isolate thousands of nuclei of each neural cell type of human caudate nucleus and putamen to generate deep, high-resolution, cell-type-specific transcriptional and *HTT* CAG repeat tract length-measurement data from control and HD donors. Our data reveal that somatic expansion of *mHTT* CAG tract occurs in select striatal neuron types. Our findings are consistent with models of HD pathogenesis in which somatic CAG expansion is a critical first step in pathogenesis, followed by a second step in which the expanded *mHTT* allele has a toxic effect that eventually leads to degeneration and death of the cell^16^. In addition, our data indicate that somatic *mHTT* CAG expansions alone may not be sufficient to explain cell-type vulnerability and reveal several cell-type-specific molecular features of the disease.

The most vulnerable cell types in the HD striatum are MSNs^2^. Although both dMSNs and iMSNs are progressively lost during the progression of the disease, iMSNs that express dopamine receptor D2 and enkephalin are most vulnerable in early stages^21^. Striatal interneurons are relatively spared early in the disease^3,5^. In particular, although CHAT^+^ INs are clearly affected, as indicated by reduced CHAT activity in histological sections, the persistent expression of acetylcholinesterase in these cells indicates that they do not die during the disease^4,48^.

Our data showing somatic *mHTT* CAG expansion in both MSNs and cerebellar PCs^24,49,50^ support the hypothesis that CAG expansion is an early step in disease progression that is necessary for the loss of neurons in HD. Accordingly, the stability of the *mHTT* CAG tract we see in SST^+^, TAC3^+^ and PVALB^+^ INs can explain their relative resilience in HD. However, our data also demonstrate that large expansions of the *mHTT* CAG tract are not sufficient for loss of CHAT^+^ INs in HD. Furthermore, data we collected from dMSN and iMSN nuclei isolated from HD donors, especially from carriers of reduced-penetrance *mHTT* alleles where the loss of MSNs is minor, establish that differences in the rate of *mHTT* CAG expansion are an unlikely explanation for the reportedly greater vulnerability of iMSNs than dMSNs in this disease^21^. The conclusion that substantial somatic expansion is required but may not be sufficient for neuronal loss in the HD brain is supported by studies of the human cerebral cortex in HD, demonstrating that extensive expansion of the *mHTT* CAG tract occurs in many deep layer pyramidal cell types despite selective loss of L5a corticostriatal projection neurons^51^.

It is important to note that the assay we have employed for determining CAG repeat lengths is limited to a tract length of 113 CAG repeats and thus cannot be used to detect the very long CAG expansions that have been reported to occur in some HD donors’ brains^12^. It remains possible, therefore, that cell loss from the HD striatum is due to CAG repeats that have undergone extremely large somatic expansion, and that the differential resilience of CHAT^+^ INs relative to MSNs during HD progression, as well as the resilience of MSNs with somatically expanded *mATXN3* repeat (Supplementary Note 6), is explained by a difference in the frequency of very long CAG repeats that are undetectable by our assay. A threshold of 115 CAGs has been postulated for striatal cell loss based on computational models of somatic expansion that include acceleration of expansion as the length of the CAG repeat increases^52^. Analysis of the genomes of cells that have already died in the HD brain, for example by recovery of MSN DNA from cerebrospinal fluid, would provide data directly addressing the threshold of CAG expansion required for cell death.

The preferential expansion of the *mATXN3* CAG tract we detect in MSN nuclei isolated from SCA3 donor samples indicates that these neurons have a general propensity to expand long CAG tracts, perhaps as a consequence of the high level of MutSβ (Supplementary Note 7). We offer mechanistic insight to how elevated MutSβ could be promoting somatic CAG expansions by showing that an excess of MutSβ inhibits FAN1 nucleolytic excision of excess CAG slip-outs, thereby allowing slip-outs to be retained as somatic expansions (Supplementary Note 8).

Our data show that HD-associated gene expression changes in human MSNs are distinct from those of other striatal neurons and have only a partial overlap to gene expression changes documented in published datasets from HD mouse models^53,54^ (Supplementary Note 9). The large magnitude of HD-associated downregulation seen for many genes (median log_2_ fold change −0.67 and −0.73 for genes downregulated in dMSNs and iMSN, respectively) indicates that extensive transcriptional disturbances occur in the majority of MSNs prior to their demise. The strong induction of genes involved in autophagic clearance argues that human MSNs mount an important defense against the mHTT misfolding and extranuclear aggregation in HD. Other changes may be more detrimental, including the strong transcriptional downregulation of genes involved in nuclear functions, perhaps as a direct effect of the presence of mHTT in the nucleus. Moreover, there are large expression changes in several genes which have been shown to be required for MSN viability in mouse models of HD^43^, and in genes with a clearly established link to human MSN function (*ANO3*) and survival (*TAF1* and *PDE10A*).

The data we have reported here strongly support previous proposals^16^ that somatic CAG expansion is a necessary first step in the pathophysiological cascade that unfolds in HD. Our data suggest also that striatal MSNs are prone to somatic CAG expansion, perhaps as a consequence of the high level of MutSβ that we have documented in MSN nuclei. It remains possible that differences in the frequency of very long *mHTT* repeats in vulnerable and more resilient cell types is sufficient to explain their differential death in the HD brain. However, given the number and magnitude of MSN-specific transcriptional responses evident in the human striatum in HD, precedent from mouse models^54^, and the nature of the specific genes dysregulated, we favor the proposal that some of these gene expression changes are an indicator and likely also a cause of compromised human MSN function well before the eventual loss of these cells in HD. We hope that further analyses of the comprehensive datasets we have provided will stimulate others to interrogate them in the context of detailed mechanistic studies to clarify the degree to which these transcriptional changes perturb the implicated biological processes in human brain, and whether these changes are detrimental or compensatory.

## Methods

### Human samples

Deidentified tissue samples analyzed in this study were determined to be exempt from Institutional Review Board review according to 45 CFR 46.102 (f). For this work, fresh frozen brain samples were obtained from Miami’s Brain Endowment Bank, University of Washington BioRepository and Integrated Neuropathology Laboratory, Columbia University Alzheimer’s Disease Research Center, The University of Michigan Brain Bank and Netherlands Brain Bank or through the National Institutes of Health (NIH) NeuroBioBank and sourced from either the Harvard Brain Tissue Resource Center or the NIH Brain & Tissue Repository-California, Human Brain & Spinal Fluid Resource Center, VA West LA Medical Center (Los Angeles, CA). Drug addiction and schizophrenia as well as clinical evidence of brain cancers were reasons for sample exclusion, whereas samples from donors with a history of other non-brain cancers and diabetes were accepted. Caudate nucleus, putamen and cerebellar vermis were used for isolation of nuclei. The brain regions used from each donor and their age, race, sex and post-mortem interval are noted in Supplementary Table 1. The table includes information about the Vonsattel grade, calculated CAP100 score^55^, the number of uninterrupted CAG repeats in their *HTT* alleles and the sequence of the CAG tract and the adjacent CCG tract, as determined from CAG tract length measurement data.

### Isolation, labeling and sorting of glial cell nuclei

Nuclei were isolated as described previously^17^. For the labeling of glial cell nuclei and cerebellar granule cells, the isolated nuclei were washed once with homogenization buffer (0.25 M sucrose, 150 mM KCl, 5 mM MgCl_2_, 20 mM Tricine pH 7.8, 0.15 mM spermine, 0.5 mM spermidine, EDTA-free protease inhibitor cocktail, 1 mM DTT, 20 U ml^−1^ SUPERase-In RNase inhibitor (ThermoFisher, #AM2696), 40 U ml^−1^ RNasin ribonuclease inhibitor (Promega, #N2515)). Each washing step constituted of resuspension of nuclei pellet followed by centrifugation (1,000 × *g*, 4 min, 4 °C). Resuspended nuclei were fixed in Homogenization buffer with 1% formaldehyde for 8 min at room temperature followed by quenching with 0.125 M glycine for 5 min. Following centrifugation, the nuclei were washed once with wash buffer (PBS, 0.05% TritonX-100, 0.5% BSA, 20 U ml^−1^ Superase-In RNase Inhibitor and 40 U ml^−1^ RNasin ribonuclease inhibitor) and incubated at room temperature on a shaker in wash buffer for permeabilization and blocking of unspecific binding. Nuclei were washed twice in wash buffer without TritonX-100 and resuspended in 100 µl 40% ethanol containing TrueBlack Lipofuscin Autofluorescence Quencher (Biotium, #23007) for 40–50 seconds. Nuclei were washed twice with wash buffer (w/o TritonX-100) and incubated overnight at 4 °C with the following antibodies: Rb x NeuN-Alexa-647 (1:400, Abcam, #ab190565), Rb x NeuN-Alexa594 (1:400, Abcam, #ab207279), Mm x EAAT1 (1:2,000, Santa Cruz Biotechnology, #sc-515839), Mm x IRF8-PE (1:65, ThermoFisher, #12-9852-82) and Goat x Olig2 (1:300, R&D Systems, #AF2418). After three washes with wash buffer (w/o TritonX-100), the nuclei were incubated for 30–45 min at room temperature with Donkey × Mm-Alexa-488 (1:1,000, ThermoFisher, #A-21202) and Donkey x Goat-Alexa-647 (1:300, ThermoFisher, # A-21447). After three washes with wash buffer (w/o TritonX-100), the nuclei were resuspended in Sorting buffer (PBS, 0.2% BSA, 40 U ml^−1^ RNasin ribonuclease inhibitor, 0.5 µg ml^−1^ DAPI) and separated with SONY MA900 Cell Sorter (software ver. 3.0.5). Aggregates of nuclei were excluded based on higher DAPI signal and the following gating strategies were used: neuronal nuclei (647+, 594+, 488−, large), oligodendrocyte nuclei (647+, 594−, 488−, small), microglia nuclei (647−, 594+, 488−, small) and astrocyte nuclei (647−, 594−, 488+, small). A separate sorting experiment was performed for collecting cerebellar granule cell nuclei. For this purpose, nuclei were labeled with Rb x NeuN-Alexa594 (1:400, Abcam, #ab207279) and Mm x ITPR1-Alexa-488 (Santa Cruz Biotechnology, #sc-271197 AF488), and granule cell nuclei were collected (488−, 594+).

For labeling neuronal nuclei, PrimeFlow labeling kit (ThermoFisher, #88-18005-210) was used and fixation and permeabilization were carried out according to manufacturer’s instructions but with 200 U ml^−1^ Superase-In RNase inhibitor and 400 U ml^−1^ RNasin ribonuclease inhibitor present at every incubation step. For sorting, the nuclei were resuspended in sorting buffer (PBS, 0.2% BSA, 40 U ml^−1^ RNasin ribonuclease inhibitor, 0.5 µg ml^−1^ DAPI). Probes specific to DRD1 (Alexa-647, #VA1-3002351-PF), DRD2 (Alexa-488, #VA4-3083767-PF) and PPP1R1B (Alexa-568, #VA10-3266354-PF) were used to label dMSN (647+, 568+, 488−, large) and iMSN nuclei (647−, 568+, 488+, large). In a separate set of experiments, probes specific to TAC3 (Alexa-647, #VA1-16603-PF), ETV1 (Alexa-488, # VA4-3083818-PF), SST (Alexa-568, # VA10-3252595-PF) and PPP1R1B (Alexa-568, # VA10-3266354-PF) were used to label the nuclei of TAC3+ interneurons (647+, 568−, 488+), PVALB+ interneurons (647−, 568−, 488+), SST+ interneurons (647−, 568+++, 488−) and MSNs (647−, 568+, 488−, large). Probes specific to TRPC3 (Alexa-647, # VA1-3004835-PF), COL6A6 (Alexa-647, #VA1-3014134-PF) and PPP1R1B (Alexa-568, # VA10-3266354-PF) were used in another set of experiments to label cholinergic interneuron nuclei (647+, 568−, large) and MSN nuclei (647−, 568+, large). CA8 probe (Alexa-647, #VA1-3001892-PF) was used for sorting Purkinje neuron nuclei (647+, large). Aggregates of nuclei were always excluded based on higher intensity of DAPI staining. All PrimeFlow target probes were used at a dilution of 1:40.

### ATAC-seq library preparation

For generating ATAC-seq data, the nuclei were treated with Tagment DNA TDE1 Enzyme (Illumina, #15027865) before fixation and labeling. The exact number of nuclei processed depended on the abundance of the population labeled and collected. Briefly, 800,000 nuclei were pelleted by centrifugation (5 min at 950 × *g*) and resuspended in 10 mM Tris-HCl pH 7.6, 10 mM NaCl, 3 mM MgCl_2_, 0.01% NP-40 followed by centrifugation (500 × *g* for 10 min at 4 °C). The pellet was resuspended in 200 μl Transposition Mix (1× TD buffer containing 20 U ml^−1^ Superase-In RNase Inhibitor, 40 U ml^−1^ RNasin ribonuclease inhibitor and 1.25 µl Illumina Tagment DNA TDE1 Enzyme per every 100,000 nuclei) and incubated at 37 °C for 30 min. The reaction was stopped and nuclei fixed by adding 1 ml homogenization buffer with 1 mM EDTA and 1% formaldehyde. After 8 min of incubation on a shaker, the fixative was quenched by adding glycine (0.125 M) for 5 min. After washing the nuclei once in homogenization buffer and once in wash buffer (without Triton X-100), the sample was processed like described above, proceeding with the steps that follow permeabilization of nuclei. After sorting, the collected nuclei were centrifuged at 1,600 × *g* for 10 min 4 °C and resuspended in 200 μl RC solution (50 mM Tris-HCl pH 7.6, 200 mM NaCl, 1 mM EDTA, 1% SDS and 5 µg ml^−1^ Proteinase K) and incubated overnight at 55 °C. Genomic DNA was isolated with using MinElute Reaction Cleanup Kit (Qiagen, #28206) and used for PCR amplification (72 °C, 5 min; 98 °C, 30 s; 12-14× (98 °C, 10 s; 63 °C, 30 s; 72 °C, 1 min)) with NEBNext High-Fidelity 2X PCR Master Mix (New England Biolabs, #M0541S) and barcoded Nextera primers (1.25 μM each)^56^. Following double-sided size selection by bead-purification the libraries were quantified with Qubit dsDNA HS assay kit (ThermoFisher #Q32851) and pooled for sequencing on NovaSeq6000 (SP 2 × 100 bp).

### FANS-seq library preparation and sequencing

RNA extraction was carried out with AllPrep DNA/RNA FFPE Kit (Qiagen, #80234) with modifications described previously^17^. RNA-seq libraries were prepared with Trio RNA-Seq library preparation kit (Tecan, #0506-A01), quantified with Qubit dsDNA HS assay kit (ThermoFisher #Q32851) and pooled for sequencing on NovaSeq6000 (SP 2 x 150 bp).

### RNA-seq data processing

Sequence and transcript coordinates for human hg38 UCSC genome and gene models were retrieved from the BSgenome.Hsapiens.UCSC.hg38 Bioconductor package (version 1.4.1) and TxDb.Haspiens.UCSC.hg38.knownGene (version 3.4.0) Bioconductor libraries, respectively. FANS-seq reads were aligned to the genome using Rsubread’s subjunc method (version 1.30.6)^57^ and exported as bigWigs normalized to reads per million using the rtracklayer package (version 1.40.6). Reads in genes were counted using the featurecounts function within the Rsubread package against the full gene bodies (Genebody.Counts) and gene exons (Gene.Counts).

### ATAC-seq data processing

The ATAC-seq reads were aligned with the hg38 genome from the BSgenome.Hsapiens.UCSC.hg38 Bioconductor package (version 1.4.1) with Rsubread’s align method in paired-end mode. Fragments between 1 and 5,000 bp long were considered correctly paired. Normalized, fragment signal bigWigs were created with the rtracklayer package. Peak calls were made with MACS2 software in BAMPE mode^58,59^. For each striatal interneuron type except cholinergic interneurons, the ATAC-seq consensus peaks were called from four ATAC-seq datasets generated from four different control donors. For MSNs, ATAC-seq consensus peaks were called from 8 dMSNs datasets from 7 different HD donors, from 9 iMSN datasets from 8 different HD donors, and 31 dMSNs datasets and 32 iMSN datasets from 8 control donors (up to four datasets from each donor). High-confidence consensus peaks were derived by creating a nonredundant peak set for each cell type and disease state and then filtering down to peaks that were present in the majority of samples. These were then annotated to TSS based on proximity using the ChIPseeker package (version 1.28.3)^60^. NCBI Refseq hg38 gene annotation was used (version 109.20211119).

### Differential gene expression analysis and principal-component analysis

For comparison of transcript abundance data between different cell types from control donors, the comparisons of data from caudate nucleus and putamen were done independently. For control donors from whom there were data available from both posterior and anterior parts of the same structure, a single table of average raw read counts per gene was generated for each cell type. For comparison of control donor data to HD donor data, up to four separate datasets for a given cell type (anterior putamen, posterior putamen, anterior caudate nucleus and posterior caudate nucleus) were combined by calculating the average raw read counts per gene (rounding up to integer), so that each donor was represented by a single FANS-seq dataset for each cell type. Principal-component analysis plots for 500 most variant genes were generated with pcaExplorer^61^ using average raw read ‘Genebody.Counts’ tables as input data (one table for each donor per cell type). Average raw read ‘Gene.Counts’ tables (that is, derived from FANS-seq reads mapped to exons), one for each donor per cell type, were converted into normalized counts by DESeq2, thereby accounting for sequencing depth differences, and used for differential gene expression analysis by DESeq2 (refs. ^62,63^) (version 1.36.0) (Supplementary Table 5; adjusted *P* < 0.05 is considered as a significant difference). Differential gene expression analysis performed based on ‘Genebody.Counts’ (that is FANS-seq reads mapped to full gene bodies) is also provided (Supplementary Table 6). Differential gene expression analysis results were filtered to exclude genes for which none of their annotated TSS positions in NCBI Refseq hg38 (version 109.20211119) overlapped with ATAC-seq consensus peaks defined separately for the cell types compared. These lists were augmented with a small number of genes (<110) for which visual inspection of mapped FANS-seq and ATAC-seq reads in Integrative Genomics Viewer^64^ suggested that these genes were in fact expressed (marked as ‘Visual inspection of mapped FANS-seq and ATAC-seq reads‘ in Supplementary Table 3). The subset of genes inspected visually was selected based on whether they were differentially expressed (that is, DEGs) between HD and control donors, the logic being that the exclusion of genes that have accessible TSS but are not DEGs would not have any effect on Gene Ontology analysis results. For the visualization of gene expression differences across cell types, ‘expression in cell type A’ was calculated as the mean of DESeq2-normalized ‘Gene.Counts’ from each donor. ‘Expression in a cell type A’ was then turned into relative expression (‘relative expression in cell type A’ = ‘expression in a cell type A’ divided by ‘mean of expression in all cell types compared’) and the resulting values were log_2_-transformed for visualization by Pheatmap R package (version 1.0.12). Relative expression was calculated in the same manner when comparing expression across individual samples instead of cell types.

### Motif analysis and annotation

High-confidence consensus peaks for MSNs were annotated to genes using the ChIPseeker package^60^ and filtered to remove ‘Distal Intergenic’ peaks. The remaining peaks were overlapped with the list of genes either up- or downregulated in HD dMSNs and iMSNs (Supplementary Table 5) to generate three peak sets for each cell type: ‘peaks in HD-upregulated genes,’ ‘peaks in HD-downregulated genes,’ and ‘peaks in genes with no expression change’. 200-bp wide DNA sequence at the center of each peak was retrieved using the BioStrings R package (2.66.0). These DNA sequences were then used as input for MEA^65^ with the following comparisons: ‘HD-Upregulated vs. No Expression Change’, ‘HD-Upregulated vs. Randomized GC-content Matched Background’ and ‘HD-Upregulated vs. HD-Downregulated’ (peaks in HD-Downregulated’ genes were similarly compared to peaks in ‘HD-Upregulated’, ‘No expression change’ and ‘Randomized GC-content Matched Background’ genes). Overrepresentation of motifs in eukaryote in vivo and in vitro databases was then calculated using a Fisher’s exact test and average odds score, where matches must have a log-odds score ≥ 0.25 times the maximum possible log-odds score. Motifs with E_value_ < 10 and *P*_adj_ < 0.005 were considered to have significant overrepresentation over the sequences in control peak sets (Supplementary Table 9). The results were visualized by *memes* R package (1.6.0, using *plot_ame_heatmap* function), displaying those motifs that were significantly overrepresented across all three comparisons (either in dMSN or iMSNs), excluding genes for which none of their annotated TSS positions overlapped with ATAC-seq consensus peaks.

### Pathway enrichment analysis

The filtered lists of DEGs (*P*_adj_ < 0.05) with accessible TSS regions were analyzed for overrepresentation of GOCC terms with enrichGO function of clusterProfiler package^66^ (version 4.4.4, GOSOURCEDATE: 2022-03-10). The augmented list of all genes with accessible TSS regions was used as the ‘background’ list for comparison (‘universe’), and the following parameters were used: qvalueCutoff = 0.05, minGSSize = 5, maxGSSize = 2000. For identifying GOCC pathways overrepresented among genes that showed disease-associated upregulation or downregulation only in dMSNs, the list of DEGs from ‘HD_dMSN vs ctrl_dMSN’ comparison (*P*_adj_ < 0.05 with DESeq2) was filtered to exclude genes that had changed expression (*P*_adj_ < 0.05 with DESeq2) in the same direction in any of the three ‘HD_interneuron vs. ctrl_interneuron’ comparisons (TAC3^+^ INs, PVALB^+^ INs or SST^+^ INs). Significance threshold for enrichment analysis: *q* value < 0.05, *P*_adj_ < 0.05, adjusted for multiple comparisons after hypergeometric test with clusterProfiler. GOCC term enrichment analysis (with parameters and ‘background’ list specified above) was also performed for genes downregulated in HD MSNs that are essential for MSN survival in wild-type mice (based on shRNA screening)^43^.

### *HTT* and *ATXN3* CAG tract sizing

Genomic DNA was purified using AllPrep DNA/RNA FFPE Kit (Qiagen, #80234) and concentrated in a vacuum concentrator if required. *HTT* CAG tract sizing was done by next generation sequencing of PCR amplicons of *HTT* exon 1 using a modified version of a previously published protocol^20^. Up to 10 ng gDNA was amplified in a 20 μl volume using NEBNext High-Fidelity 2X PCR Master Mix (New England Biolabs, #M0541S) supplemented with 5% dimethyl sulfoxide and barcoded primers specific to *HTT* exon 1 (0.5 μM each)^20^ or *ATXN3* exon 10 (primer sequences in Supplementary Table 12): 1 cycle 96 °C, 5 min; 30× [96 °C, 45 s; 61 °C, 45 s; 72 °C, 3 min]; 72 °C, 10 min. The number of amplification cycles was raised to 32 cycles if the amount of gDNA input was below 4 ng. After PCR, the samples were combined into small pool of two to six samples and size selection was carried out by adding 0.55× volume of AMPure XP beads (Beckman Coulter, #A68831). The concentrations of purified library pools were quantified with Collibri Library Quantification Kit (ThermoFisher, #A38524100), combined into a sequencing library and sequenced on MiSeq sequencer using a 500 cycle MiSeq Reagent Nano Kit v2 with both index reads, but with 400-nt long read 1 and no read 2. Demultiplexed sequencing read data was aligned using Burrows-Wheeler Aligner (https://github.com/lh3/bwa, using BWA MEM default settings except: -O 6,6 -E 4,4) to a set of *HTT* exon 1 or *ATXN3* exon 10 reference sequences (Supplementary Data 1 and 2) that differed by the number of CAG repeat units in the repeat tract. The number of reads uniquely mapped to each of the reference sequences in the set was considered to reflect the distribution of CAG tract lengths in the two *HTT* or *ATXN3* alleles in the cell population analyzed. *HTT* read mapping data from each donor was inspected manually for determining the nucleotide sequence of the adjacent polyproline tract and the presence/absence of interruptions in CAG tract. If *mHTT* exon 1 structure of the donor was atypical, then sequencing reads were realigned to a set of reference sequences matching that *mHTT* exon 1 structure. The length of CAG repeat tracts reliably mapped was limited to 113 repeat units. Uninterrupted CAG tract lengths of progenitor/unexpanded *mHTT* allele (M repeat units) and normal *HTT* allele (N repeat units) were defined from the two modes of mapped read length-distribution in CAG-sizing data from non-expanding cell types (usually striatal microglia and astrocytes, or, if available, cerebellar granule cells). R is the number of reads mapped to a reference sequence with the specified CAG tract length. The ratio of somatic expansions (RoSE)^20^ and mean somatic length gain (MSLG, measured in repeat units (RUs)) were calculated as follows:

$${\mathrm{normal}}\,{\mathrm{allele}}\,{\mathrm{RoSE}}=\frac{{R}_{N+1}+{R}_{N+2}+{R}_{N+3}}{{R}_{N}}$$

$${\mathrm{mutant}}\,{\mathrm{allele}}\,{\mathrm{RoSE}}=\frac{\mathop{\sum }\nolimits_{i=M+1}^{113}{Ri}}{{R}_{M}}$$

$${\mathrm{Mean}}\,{\mathrm{somatic}}\,{\mathrm{length}}\,{\mathrm{gain}}\,\left({\mathrm{RU}}\,\right)=\frac{\mathop{\sum }\nolimits_{i=M}^{113}\left({Ri}\times i\right)}{\mathop{\sum }\nolimits_{i=M}^{113}{Ri}}-M$$

Mean somatic length gain is the average uninterrupted CAG repeat length in sequencing reads from which the progenitor allele CAG repeat length (M) has been subtracted. It is important to note that the term is not meant to reflect the size of incremental change per mutation event. Quantification of CAG tract length changes for *mATXN3* was done in the same way. Statistical analysis of differences between cell types was carried out by comparing their ratio of somatic expansions or mean somatic length gains with one-way ANOVA, followed by Holm–Sidak’s multiple comparisons test.

### Western blotting

When isolating nuclei for western blotting the tissue homogenization and ultracentrifugation steps were carried out as described by Xiao et al.^17^. After washing the nuclei once in homogenization buffer the nuclei were resuspended in 1 ml 1× PBS, 0.05% Triton X-100, 2% BSA and incubated at room temperature on a shaker for ~15–20 min. The nuclei were labeled by adding the following antibodies: Rb x NeuN-Alexa-647 (1:300, Abcam #ab190565), Rb x NeuN-Alexa594 (1:300, Abcam, #ab207279), Mm x EAAT1-Alexa-488 (1:200, Santa Cruz Biotechnology, #sc-515839 AF488), Mm x IRF8-PE (1:65, ThermoFisher, #12-9852-82) and Goat x Olig2 (1:200, R&D Systems, #AF2418). After two washes with WB wash buffer (1× PBS, 0.05% Triton X-100, 0.2% BSA), the nuclei were incubated for 30 min at room temperature with Donkey x Goat-Alexa-647 (1:400, ThermoFisher, # A-21447). Nuclei were washed twice with WB wash buffer and resuspended in Sorting buffer (w/o RNase inhibitors). After sorting, the collected nuclei were centrifuged at 1,600 × *g* for 10 min 4 °C and the residual volume was kept to a minimum. Nuclei were treated with DNase I (in the presence of 0.5 mM MgCl_2_) at 37 °C for 10 min, mixed with NuPAGE Sample Reducing Agent (ThermoFisher #NP0004) and β-mercaptoethanol (final concentration 4%, Sigma, #M3148), and heat-denatured at 96 °C for 3 min. Material from 25,000 to 50,000 nuclei were loaded on NuPAGE 4% to 12% Bis-Tris Mini gels (ThermoFisher, #NP0322BOX), aiming for equal loading in each well. After blotting the samples onto nitrocellulose membrane and blocking unspecific binding by incubating the membrane in a 5% solution of non-fat dry milk, the membranes were probed with Rb × Histone H3 antibody (1:5,000, Abcam, #ab1791) and Mm × Human MSH2 (1:300 BD Biosciences, #556349) or Mm x Human MSH3 (1:300 BD Biosciences, #611390) by incubating overnight at 4 °C. After three washes with TBS-T (1× TBS, 0.1% Tween-20), the membranes were probed with IRDye 680LT Donkey anti-Rabbit IgG Secondary Antibody (1:10,000, LICOR, #926-68023) and IRDye 800CW Goat anti-Mouse IgG Secondary Antibody (1:10,000, LICOR, #926-32210) by 1 h at room temperature. After three washes with TBS-T, the membranes were imaged with Odyssey CLx Imaging System.

### Protein purification

Recombinant human FAN1 protein was expressed from a Baculovirus and purified from Sf9 insect cells as described previously^40,67^. Recombinant human MutSα (MSH2-MSH6) and MutSβ (MSH2-MSH3) were generated from Sf9 insect cells using Baculoviruses expressing his-tagged hMSH2, hMSH3 and hMSH6, and a purification procedure described previously^68–70^.

### FAN1 nuclease assay

FAN1 nuclease assays were performed as described^40^ in nuclease assay buffer (50 mM Tris-HCl pH 8.0, 25 mM NaCl, 1 mM MnCl_2_, 1 mM dithiothreitol, 200 mg ml^−1^ BSA) with 100 nM of fluorescently labeled DNA incubated with 50 nM of recombinant human FAN1 protein. Reactions were initiated by the addition of FAN1 protein, incubated at 37 °C for 20 min and then stopped with formamide loading buffer (95% formamide, 10 mM EDTA). Reaction products were electrophoretically resolved on 6% denaturing sequencing gel for 1 h at 2,000 V and detected at fluorescence filter in the Typhoon FLA (GE Healthcare). Nuclease activity quantification compared the densitometric intensity of cleaved versus uncleaved DNA (ImageQuant). In some of the experiments, different incubation time and concentration of proteins are used and are mentioned in respective figure legends. Sequences of oligonucleotides used to generate slipped-DNA substrates with anchored flanks can be found in Supplementary Note 10.

### Reporting summary

Further information on research design is available in the Nature Portfolio Reporting Summary linked to this article.

## Online content

Any methods, additional references, Nature Portfolio reporting summaries, source data, extended data, supplementary information, acknowledgements, peer review information; details of author contributions and competing interests; and statements of data and code availability are available at 10.1038/s41588-024-01653-6.

### Supplementary information

Supplementary Information Supplementary Notes 1–10 and Supplementary Figures 1–9.

Reporting Summary

Supplementary Data 1 *HTT* exon 1 reference sequences.

Supplementary Data 2 *ATXN3* exon 10 reference sequences.

Supplementary Table Supplementary Tables 1–12.

### Source data

Source Data Fig. 5 Unprocessed images of western blots.

## Data Availability

All sequencing datasets generated as part of this study are publicly available in NCBI GEO under accession GSE227729 (https://www.ncbi.nlm.nih.gov/geo/query/acc.cgi?acc=GSE227729). Further information and requests for resources and reagents should be directed to the lead contact, N. Heintz (heintz@rockefeller.edu). Altered expression of mouse genes in the striatum of BAC-CAG mice^54^ (10.1016/j.neuron.2022.01.006), the Str266R gene set^71^ (10.1101/2022.02.04.479180), list of genes essential for MSN survival in wild-type mice^43^ (10.1016/j.neuron.2020.01.004) and TRAP data from zQ175 and R6/2 mice^41^ (GEO dataset GSE152058, 10.1016/j.neuron.2020.06.021) have been published before. Sequence and transcript coordinates for human hg38 UCSC genome and gene models were retrieved from the BSgenome.Hsapiens.UCSC.hg38 Bioconductor package (version 1.4.1) and TxDb.Haspiens.UCSC.hg38.knownGene (version 3.4.0) Bioconductor libraries (https://bioconductor.org/packages/release/data/annotation/html/BSgenome.Hsapiens.UCSC.hg38.html). NCBI Refseq hg38 gene annotation (version 109.20211119, https://www.ncbi.nlm.nih.gov/genome/annotation_euk/Homo_sapiens/109.20211119/) was used for annotating ATAC-seq consensus peaks to transcriptional start sites. Gene Ontology Cellular Compartment (GOCC) terms for enrichment analysis were derived through enrichGO function of clusterProfiler package (version 4.4.4, GOSOURCEDATE: 2022-03-10, https://bioconductor.org/packages/release/bioc/html/clusterProfiler.html). Eukaryote in vivo and in vitro databases were accessed through MEME Suite 5.5.4 (https://meme-suite.org/meme/tools/ame). Source data are provided with this paper.

## Code availability

Only publicly available tools were used in data analysis. The analysis parameters used have been described in Methods.
