# Supplementary material for: Cell-type-specific CAG repeat expansions and toxicity of mutant Huntingtin in human striatum and cerebellum
Source: Nat Genet. 2024 Jan 30;56(3):383–94. doi: 10.1038/s41588-024-01653-6 (PMC10937393; doi:10.1038/s41588-024-01653-6)
Supplement: Supplementary file 1 — Supplementary Notes 1–10 and Supplementary Figures 1–9. [file 41588_2024_1653_MOESM1_ESM.pdf]

nature genetics

Article https://doi.org/10.1038/s41588-024-01653-6

Cell-type-specific CAG repeat expansions and toxicity of mutant Huntingtin in human striatum and cerebellum

In the format provided by the authors and unedited

Supplementary Notes

Supplementary Note 1.

Promoter accessibility as a measure of gene activity

Given the recent demonstration that ambient RNA released from the cytoplasm or nucleus is a source of contamination in single nucleus RNA-seq studies of brain tissue[1] and the likelihood that low-level ambient RNA can confound studies of differential gene expression, we used promoter accessibility as an independent measure of active genes[2]. To this end, we excluded genes for which none of their annotated transcriptional start sites (TSS) overlapped with regions of accessible chromatin in that cell type, as defined by the presence of a consensus peak in ATACseq data generated from the isolated nuclei (**Fig. 1d and Supplementary Table 3,** please see section '*ATACseq Data Processing*' Methods). Based on the criterion of promoter accessibility, the average number of genes expressed in each striatal cell type is ~13,000 – 14,000 (**Extended Data Fig. 2c**).

Supplementary Note 2.

Instability of *mHTT* CAG tract in striatal oligodendrocytes

To reveal differences in the "non-expanding" striatal cell types, we calculated their ratios of somatic expansion - a metric that has been used for describing the distribution of CAG tract sizes in cells undergoing modest somatic repeat expansion[3]. These data demonstrated that striatal oligodendrocytes had consistently greater CAG expansions compared to microglia, astrocytes, and SST+, PVALB+ and TAC3+ INs (**Extended Data Fig. 3b**). There was no evidence of somatic expansion of the normal *HTT* allele CAG repeat tract in any of the cell types analyzed (**Extended Data Fig. 3c**).

Supplementary Note 3.

Expression of HD age at onset modifier candidate genes in human striatum

We also studied the expression pattern of the remaining genes that were identified as candidates potentially modulating HD age at onset[4]. As expected, we found that candidate genes involved in MMR (marked with an asterisk on **Fig. 5c and Extended Data Fig. 4a**), as well as those lacking an obvious association with DNA repair are expressed at significant levels in human MSNs **(Supplementary Fig. 3a,b**). Notable exceptions are *RRM2B* and *SYT9* which are expressed at very low levels in MSNs, but at higher levels in microglia (*RRM2B*) and oligodendrocytes (*SYT9*) in both caudate nucleus and putamen **(Supplementary Fig. 3a,b,d**). While this expression pattern could not have been predicted from published cell type-specific gene expression analysis of mouse striatum[5] **(Supplementary Fig. 3c**), it is supported by corresponding differences in chromatin accessibility at the promoter regions of human *RRM2B* and *SYT9* **(Supplementary Fig. 3e**). Although these data will need to be verified by further experimentation, our data suggests that genetic variants near these genes may act in glia to

influence disease onset through mechanisms that do not impact somatic expansion of the *mHTT* CAG tract.

Supplementary Note 4

Putative transcriptional regulators of HD-associated gene expression changes

Having mapped accessible sites in the chromatin of MSNs, we used Motif Enrichment Analysis[6] (MEA) to ask whether the over-representation of transcription factor-binding motifs within genes with HD-associated expression change would predict the involvement of specific transcription factors (TFs) in the regulation of these transcriptional changes. Of the binding-motifs over-represented in genes up- and downregulated in HD MSNs (**Supplementary Table 9, Supplementary Fig. 6a**), we identified those for which the respective TF gene transcript was present at a high level in MSNs and exhibited a disease-associated change in its level. This approach led to the identification of KLF5, KLF7, ZFP14, SP3, ZKSCAN5, ZNF93, MEF2D and NFATC2 (**Supplementary Fig. 6b**) as candidate regulators for which the direction of motif over-representation (either in up- or downregulated genes) matches what is known about their function (transcriptional activator, repressor or both) and HD-associated transcript level change (up- or downregulated in HD). Interestingly, the absence of NFATC2 has been reported to increase striatal mHTT aggregate load and exacerbate neurological symptoms of HD model mice[7], thus pointing to the possibility that reduced *NFATC2* levels contribute to the dysfunction of human MSNs in HD.

Supplementary Note 5

Downregulation of genes essential for MSN viability in HD mouse models

We also asked whether any of the genes previously shown to preserve MSN viability in mice expressing *mHTT* with extra-long CAG tract[8] were strongly downregulated in HD MSNs. **Extended Data Fig. 6d** depicts genes that were found to be essential for MSN viability in the zQ175 and R6/2 mouse models, and had undergone the largest expression level decreases in human MSNs in HD donors. We consider these genes as candidates for further investigation as they can help to identify molecular processes that may contribute to loss of MSN viability in HD. Among these, HD-associated downregulation of TATA-binding protein-associated factor 1 gene (*TAF1*) in MSNs is an example of a change that could be detrimental to human MSN survival, as reduced expression and aberrant splicing of *TAF1* is thought to be the cause of MSN loss seen in X-linked dystonia-parkinsonism[9-11]. See also **Supplementary Figure 7.**

Supplementary Note 6

Expansion of *mATXN3* CAG tract in striatal MSNs

Mouse models of HD and SCA3[12-15], like humans with HD or SCA3[16-20], show age-dependent somatic CAG expansions in the CNS and peripheral tissues. Brain region patterns of CAG expansions are similar, yet mice show limited levels of degeneration. It is evident from our data that in MSNs the expansion of the *mHTT* CAG tract (mean somatic length gain approx. 22 repeat units, with repeats 113 CAG units long detected in all MSN samples from donors carrying fully penetrant *mHTT* alleles) is more extensive than the expansion of the *mATXN3* CAG tract, as very few *mATXN3* allele copies had gained more than 20 repeat units in MSNs (**Fig. 5a,** mean somatic length gain approx. 5 repeat units, and therefore only extending over the size range observed for inherited *mATXN3* alleles). These data demonstrate that in addition to the CAG repeat tract length there are other locus-specific properties that are important for the dramatic expansion of the *mHTT* CAG tract. Nevertheless, despite expansion of *mATXN3* CAG tract to over 80 CAG repeats, we did not observe a clear decrease in the number of MSN nuclei isolated from SCA3 donor samples, a finding that is consistent with neuroanatomical studies reporting MSN loss in SCA3 to be variable[21]. This reinforces the interpretation that the presence of a very long CAG repeat tract in RNA, or polyQ domain in any protein, does not necessarily lead to cell loss in the human brain. These considerations place additional emphasis on proteins that interact with mHTT *in vivo*[22], and the cell type-specific expression profiles we have made available in this study will help to delineate the cell type-specificity of these interactions in order to understand their role in HD pathogenesis.

Supplementary Note 7

Possible trans-acting factors involved in somatic expansion

Our results are suggesting that there are *trans*-acting factors that are rate-limiting in the process of repeat expansion or stabilization, and that their level of expression or activity differs between MSNs and cell types where both *mHTT* and *mATXN3* CAG tracts are more stable. In light of previously published reports on the effect of Msh2 and Msh3 in somatic CAG expansion in HD mouse models[23-26], our finding that levels of both *MSH2* and *MSH3* are higher in MSNs compared to other striatal cell types suggests that the actions of the MutSβ complex at the *mHTT* locus may be a rate-limiting step that contributes significantly to the selective vulnerability of MSNs.

We do note that our observations on elevated levels of *MSH2* and *MSH3* in MSNs do not rule out the presence of other factors that favor the expansion of *mHTT* CAG repeat tract in MSNs and CHAT+ INs. However, none of the candidate genes for modulating HD age at onset or genes influencing somatic CAG expansion in HD mouse models[27] stood out in the transcriptional profiles of CHAT+ IN nuclei as

clear candidates for facilitating somatic CAG expansion in this cell type. We note that not all factors involved in somatic repeat expansions are known, as novel factors, including primate-specific ones, are still being reported[28].

Supplementary Note 8

High MutSβ levels inhibit cleavage of slipped-CAG DNA substrates by FAN1

Our data show that an excess of MutSβ inhibits FAN1 nucleolytic excision of excess CAG slip-out DNA substrates, putative intermediates of expansion mutations. In contrast, MutSα, a complex of MSH2 with MSH6, does not affect FAN1 excision of slip-outs, consistent with a lack of involvement of MutSα in somatic expansions[24]. While both MutSα and MutSβ bind to heteroduplex DNAs, MutSβ has a very different binding mode, stronger affinity, and longer protein-loop DNA lifetimes than MutSα[29-31], and hydroxy-radical footprinting of MutSβ on CAG slip-outs showing extensive protection of the slip-out[32,33]. Excess levels of FAN1 could partly overcome the extended dwelling time of MutSβ on a CAG slip-out[29,31], resulting in slip-out excision so as to avoid retention of the excess repeats. Therefore, our results also present a possible mechanistic explanation of how genetic variants that increase FAN1 expression can delay onset of HD[34]. While mechanistic studies performed *in vitro* are limited to assessing the effects of only some of the proteins putatively involved, the model where slip-out DNA excision rates are determined by competitive binding to either MutSβ or FAN1 is appealing because the expression of *MSH2* and *MSH3*, unlike that of other repair proteins' genes, is consistently higher in MSNs when compared to any striatal interneuron type where the CAG repeat is stable, and this expression level difference stands out because of its magnitude as well.

Supplementary Note 9

Distinctions between mouse and human transcriptional responses in HD MSNs.

Analyses of gene expression changes in HD mouse models carrying human *mHTT* alleles of different CAG repeat sizes have documented a wide variety of transcriptional alterations, and there is strong evidence that these transcriptional changes are responsible for behavioral phenotypes in the absence of striatal cell loss[35,36]. While extensive transcriptional dysregulation that accompanies somatic expansion of *mHTT* CAG tract in MSNs is generally consistent with the "transcriptionopathy" that has been reported in HD mouse models[35,36], roughly only a quarter of individual genes reported as altered in the striatum of BAC-CAG mouse model are exhibiting an HD-associated expression change in human MSN FANSseq data[36] (**Supplementary Fig. 8a)**. There is a similar degree of overlap even when HD-associated changes are compared to those genes that are most consistently altered in the striatum of HD mouse models - the striatal HD signature genes[37] (**Supplementary Fig. 8b,**

Supplementary Table 11). Conversely, most of the HD-associated nuclear transcript level changes seen by FANSseq are not recapitulated in HD mouse models for which MSN-specific RNAseq data is available (**Supplementary Fig. 9a,b**). Nevertheless, downregulation of some of the MSN-enriched striatal HD signature genes (*PDE10A, ANO3, PTPN7, PCP4, RGS9, ARPP21*) that has been reported in HD mouse models takes place in human MSNs in HD as well (**Supplementary Table 11**), representing an HD-associated process that is recapitulated in mouse models of this disease[36-38].

In summary, the comprehensive transcriptional profiles we have generated from striatal cell types show that HD associated changes in MSNs have only a partial overlap to gene expression changes documented in published datasets from HD mouse models. Although it is difficult to directly compare the human FANSseq data with transcriptional changes occurring in bulk RNAseq data from the striatum or MSN translational profiling (TRAP) data from HD mouse models, the apparent discrepancies between molecular events occurring in mouse model systems and human HD donor samples place a strong emphasis on the importance of human data when initiating focused studies of candidate mechanisms of HD pathogenesis.

Supplementary Note 10

Oligonucleotides used for the generation of slip-out DNA substrates in the context of anchored duplex flanks.

Name	Oligonucleotides	Length (nt)
∆(CAG)0	5’-ATGCA $(CAG)_{10}$ ATCGT-3’ FAM	40
∆(CAG)2	5’-ATGCA $(CAG)_{12}$ ATCGT-3’ FAM	46
∆(CAG)4	5’-ATGCA $(CAG)_{14}$ ATCGT-3’ FAM	52
∆(CAG)8	5’-ATGCA $(CAG)_{18}$ ATCGT-3’ FAM	64
∆(CAG)14	5’-ATGCA $(CAG)_{24}$ ATCGT-3’ FAM	82
CTG-Bottom	5’-ACGAT $(CTG)_{10}$ TGCAT-3’	40

Note: CTG-bottom is used to make all the anchored slipped-DNA.”

Supplementary Figure 1

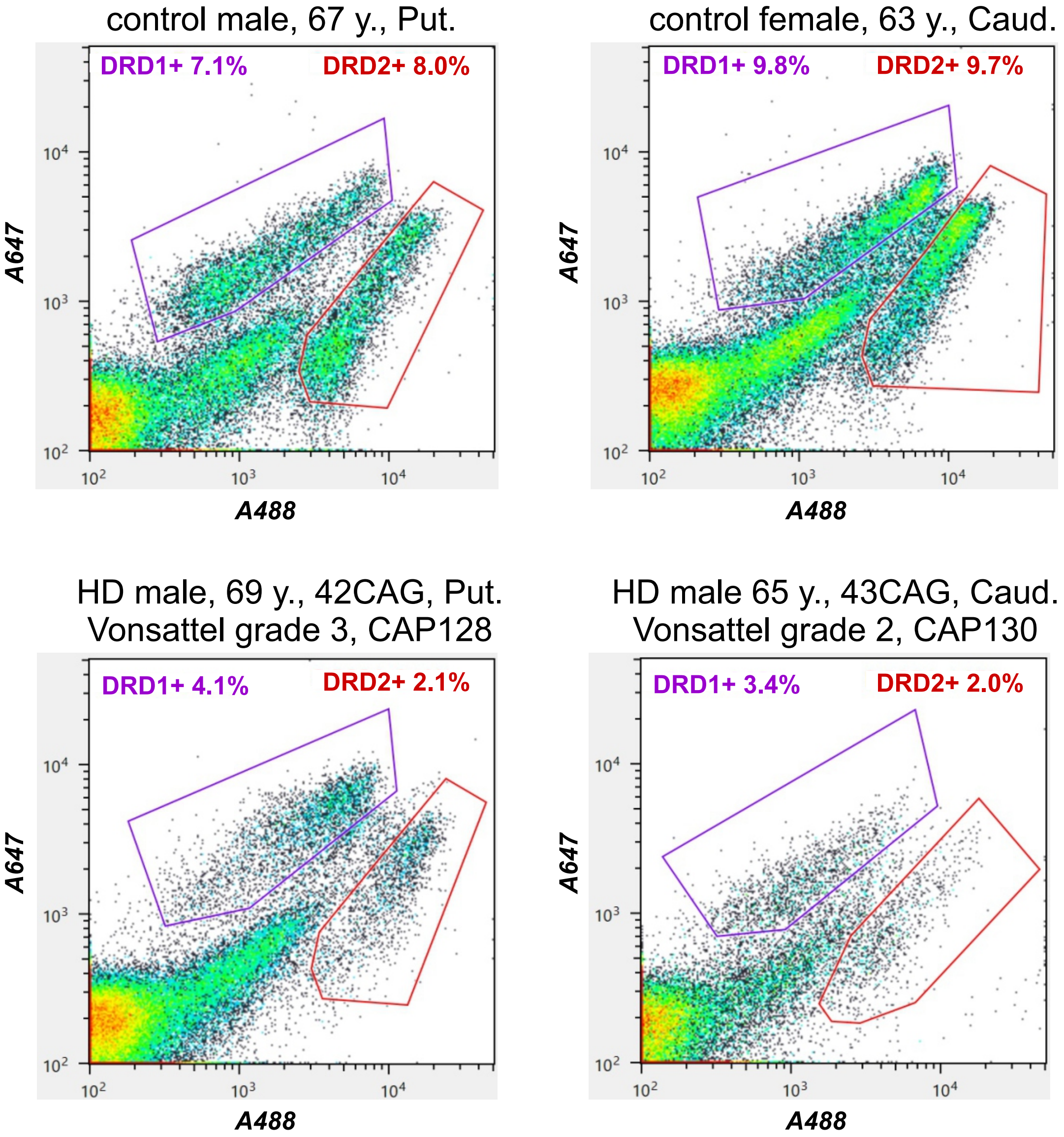

Supplementary Figure 1. Representative FANS plots showing the labeling of dMSN and iMSN nuclei with Primeflow probes specific for *DRD1* and *DRD2* transcripts, respectively. Note that while there is a clear reduction in the abundance of MSN nuclei in striatal tissue from HD donors, these probes can still be used for the separation and isolation of MSN subtypes.

Supplementary Figure 2

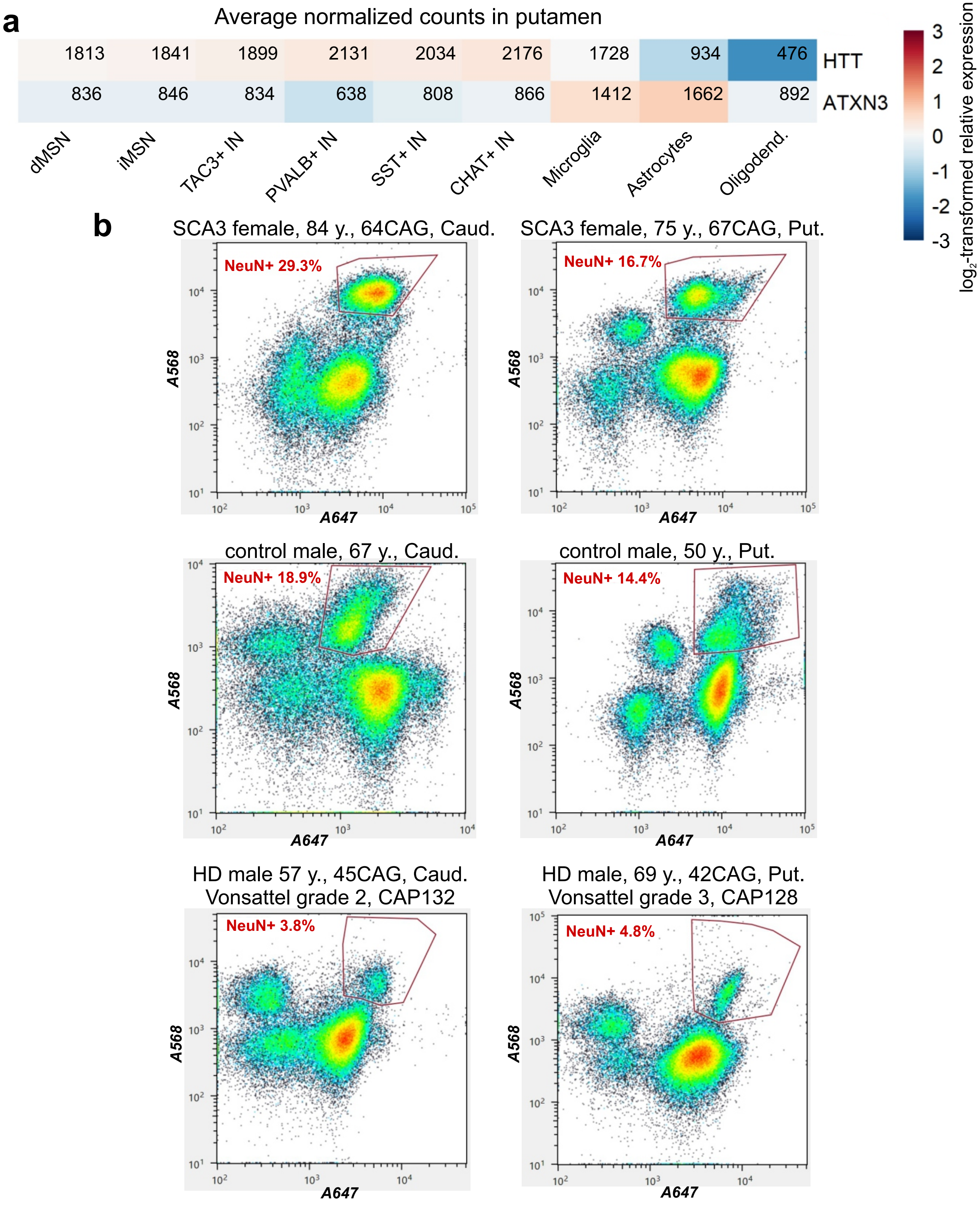

Supplementary Figure 2. **a**, Relative expression level of *HTT* and *ATXN3* in cell types of the putamen. Heatmaps depict log2-transformed relative expression in each cell type, calculated based on the mean of DESeq2-normalized counts from 6-8 control donors. **b,** FANS plots showing the percentage of nuclei stained with anti-NeuN antibody in two oldest SCA3 donors, and in two representative HD donors and control donors.

Supplementary Figure 3

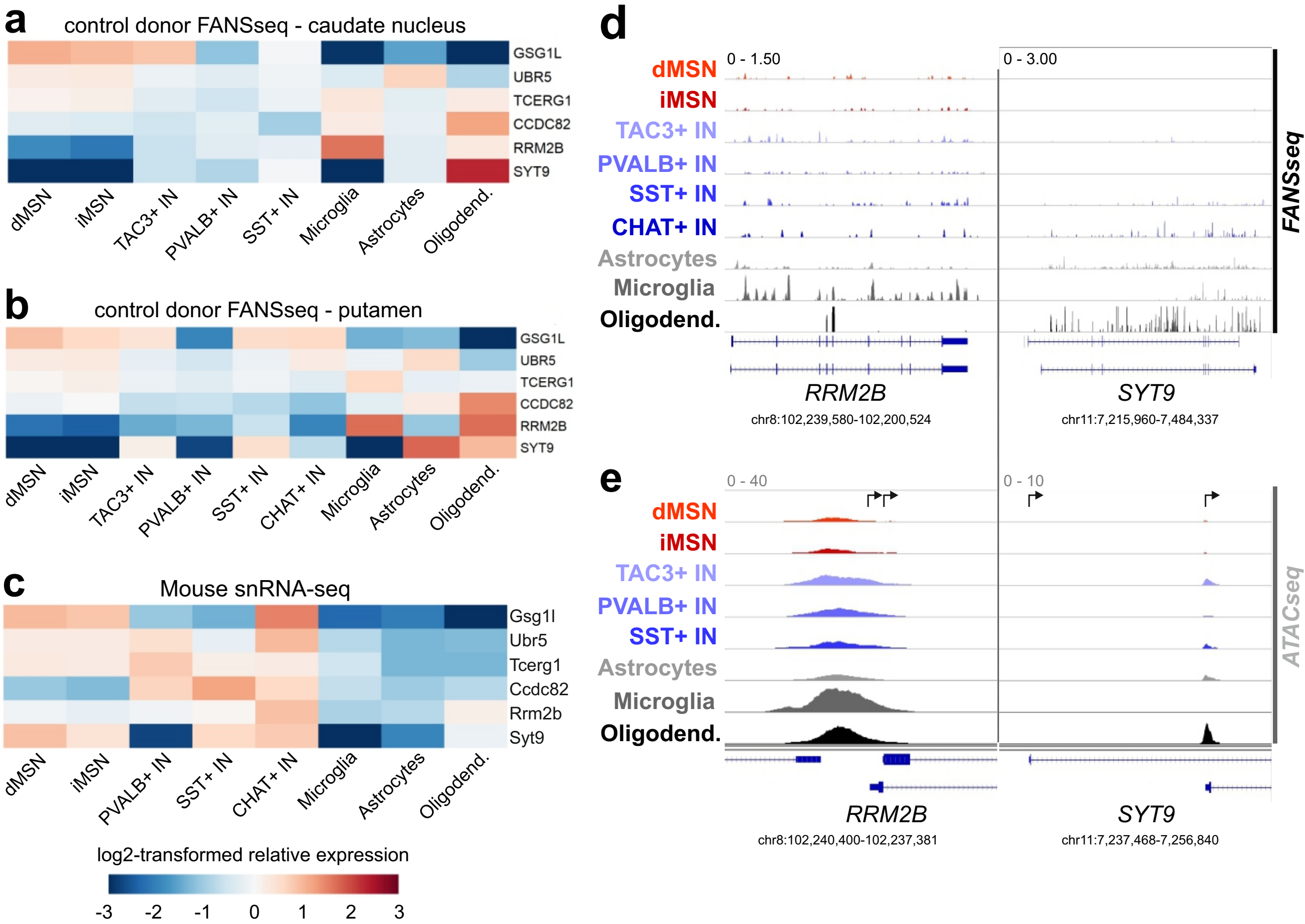

Supplementary Fig. 3. a and **b,** Relative expression level of HD age-at-onset modifying candidate genes unrelated to DNA repair. Heatmaps depict log2-transformed relative expression in each cell type of (**a**) caudate nucleus and (**b**) putamen, calculated based on the mean of DESeq2-normalized counts from 6-8 control donors. **c,** Cell type-specific expression according to snRNA-seq data from mouse striatum[5], calculated from 'transcripts per 100,000' values. **d,** Representative distribution of human FANSseq and **e,** ATACseq reads mapped to *RRM2B* and *SYT9* genes. Arrows mark the position of annotated transcriptional start sites. The data are from a 41-year-old male control donor.

Supplementary Figure 4

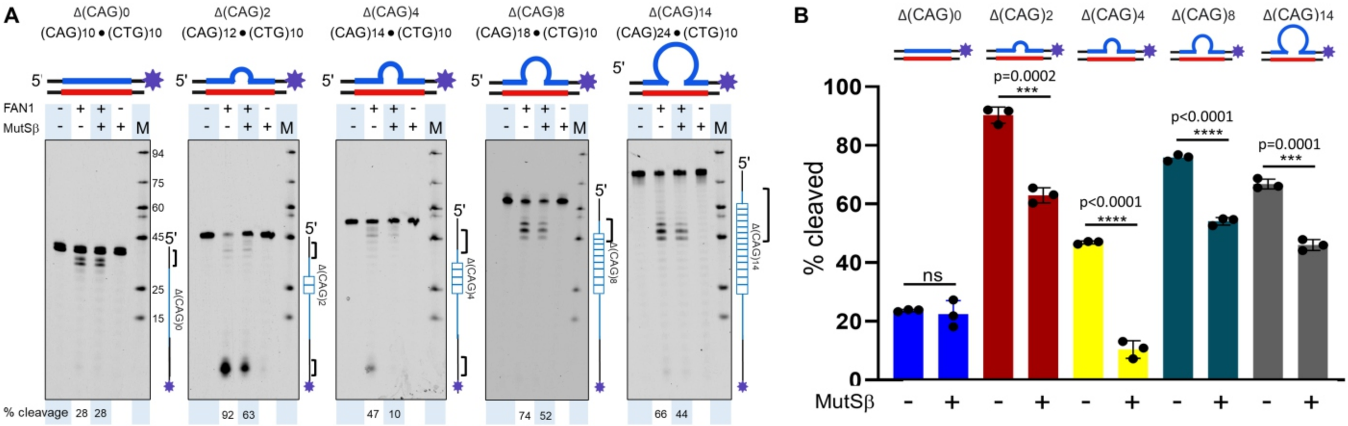

Supplementary Fig. 4. MutSβ inhibits FAN1 nuclease activity on an anchored slipped-DNA with variable amounts of excess repeats. **a,** 3'-FAM-labelled CAG-slipped DNA substrates had either 0, 2, 4, 8, or 14 excess CAG repeats on one of the strands and were anchored with 20 base pairs on each end (schematics are shown). 100 nM substrate DNAs were pre-incubated with 0 nM or 200 nM of MutSβ, reactions were initiated by adding 50 nM FAN1 and stopped 20 minutes later by adding 95% formamide-EDTA stop buffer. Reaction products were resolved on a 6% denaturing PAGE, fluorescent signals visualized by Typhoon FLA-9500, and nuclease activity quantified by comparing the intensity of the cleaved versus un-cleaved DNA, by ImageQuant. FAN1 cleavage sites are indicated by brackets in the schematics to the right of each gel. Notably, cleavage only occurred within the repeat for slip-outs of >8 excess repeats, in agreement with recent findings[39]. **b,** Quantification results for FAN1 cleavage products in absence and presence of MutSβ are plotted on graph (mean ± SD, N = 3 replicate experiments). Two-sided unpaired t-test was used for the comparison of cleavage in the presence vs absence of MutSβ (p=0.0002 (***), p<0.0001(****)).

Supplementary Figure 5

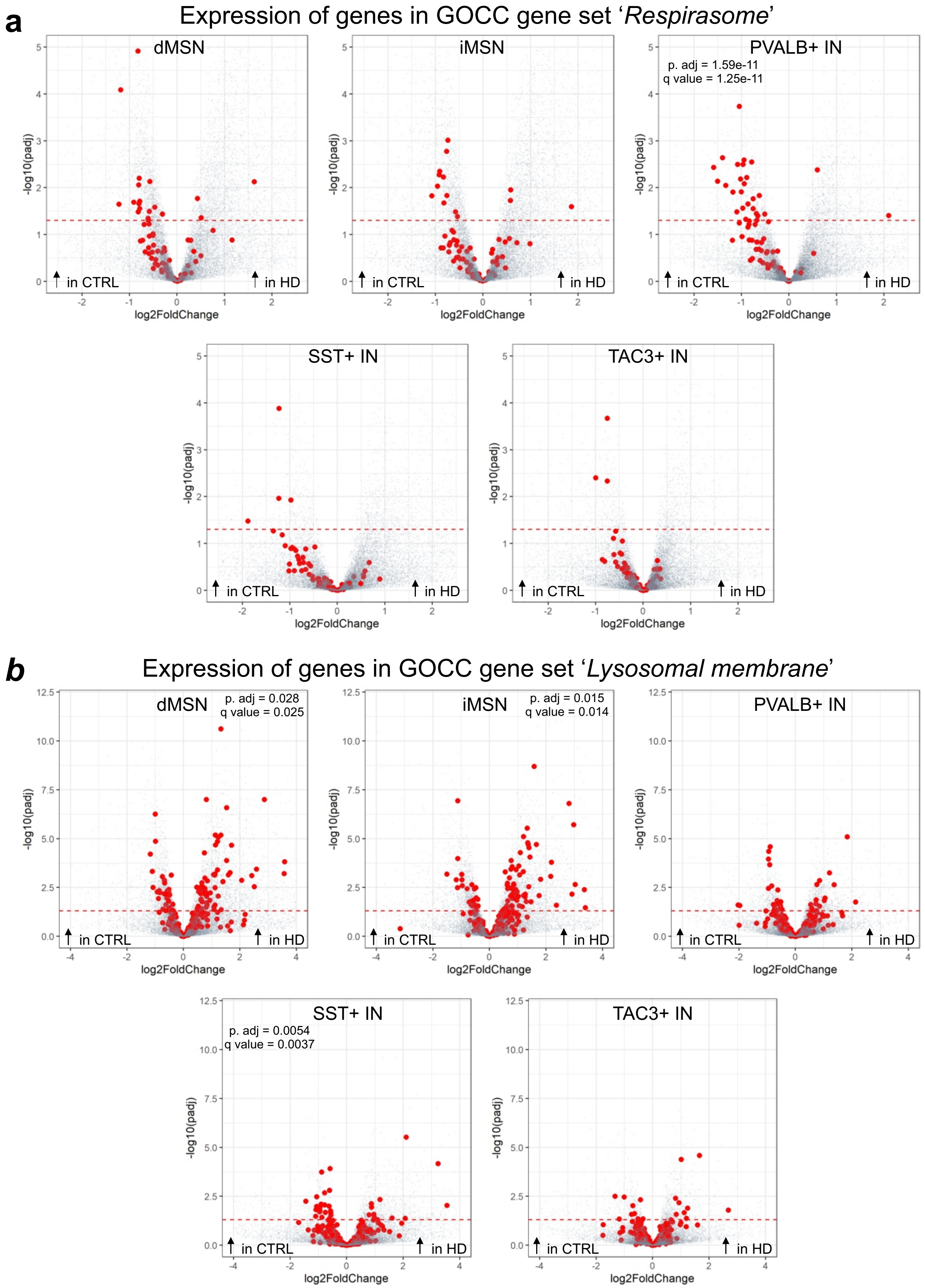

Supplementary Fig. 5. Disease-associated changes in genes associated with GO Cellular Component terms (**a**) 'Respirasome' (GO:0070469) and (**b**) 'Lysosomal membrane' (GO:0005765). For statistically significant enrichments both q and P adj. values are shown (calculated by hypergeometric test using the enrichGO function of clusterProfiler package, adjusted for multiple comparisons). Dashed line denotes the threshold of statistical significance for differential expression of individual genes (P adj = 0.05 by DESeq2).

Supplementary Figure 6

a

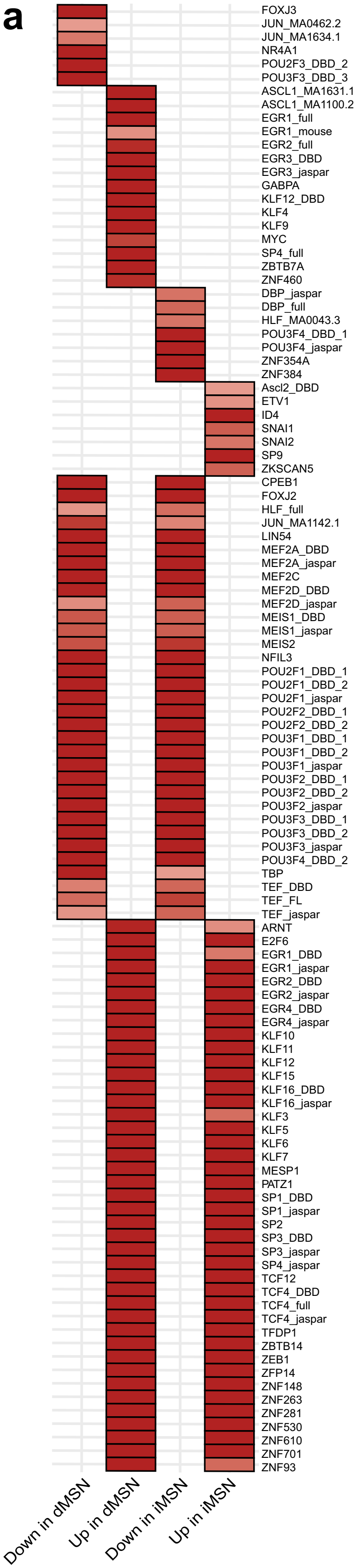

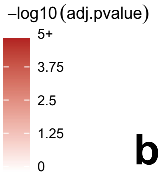

b

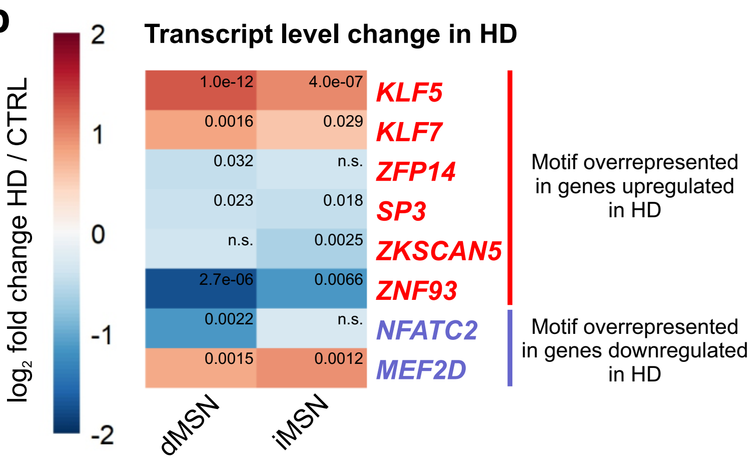

Supplementary Fig. 6. a, Transcription factor binding motifs over-represented in MSN chromatin accessible sites in genes that exhibit HD-associated transcript level increases or decreases in MSNs (see Methods). Heatmap depicts adjusted P values calculated using Fisher's Exact Test for motif enrichment analysis (adjusted for multiple comparisons). Motifs of transcription factors not expressed in MSNs are excluded (based on the inaccessibility of their TSS in MSNs). **b,** Selected transcription factors with HD-associated transcript level change and binding motif enrichment in genes either up- or down-regulated in MSNs (denoted with red and blue gene name labels, respectively). The P values indicated on heatmap represent statistical significance of transcript level differences calculated with DESeq2 and are adjusted for multiple comparisons (n=7 individuals with HD and n=8 control individuals). See also **Supplementary Note 4.**

Supplementary Figure 7

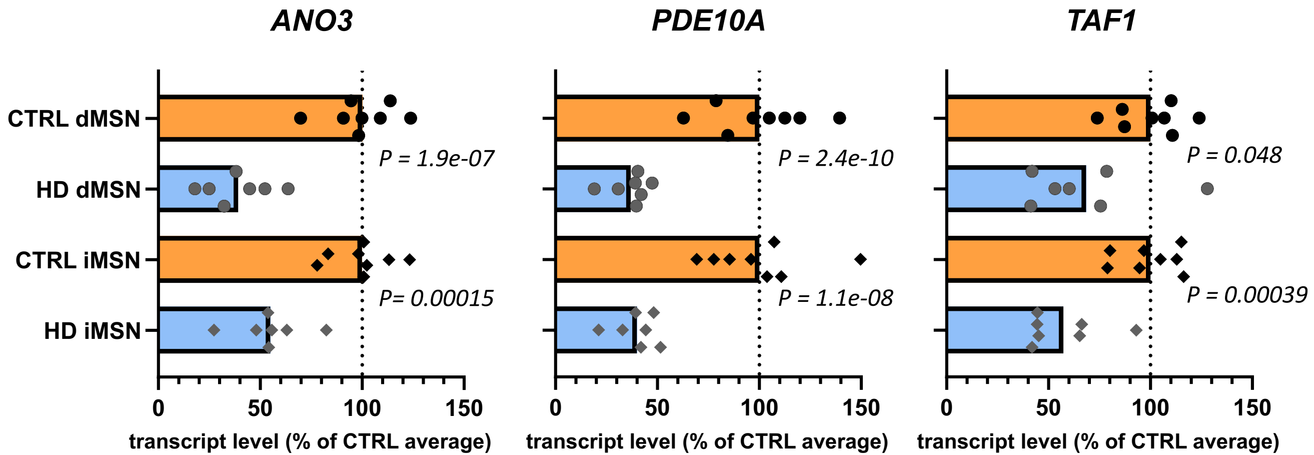

Mutations known to cause autosomal dominant form of focal dystonia (DYT24, https://omim.org/entry/615034).

Mutations known to cause dyskinesia and striatal degeneration (https://omim.org/entry/616921, https://omim.org/entry/616922).

Reduced expression and aberrant splicing of *TAF1* is thought to be the cause of MSN loss seen in X-linked dystonia-parkinsonism[9-11.] See Supplementary Note 5.

Supplementary Fig. 7. Transcript levels of *ANO3*, *PDE10A* and *TAF1* in HD MSNs represented as % of their transcript level in control donor MSNs (mean plotted). n=7 individuals with HD and n=8 control individuals. P values were calculated with DESeq2 and are adjusted for multiple comparisons: P=1.9e-07 for *ANO3* in dMSNs, P=0.00015 for *ANO3* in iMSNs, P=2.4e-10 for *PDE10A* in dMSNs, P=1.1e-08 for *PDE10A* in iMSNs, P=0.048 for *TAF1* in dMSNs and P=0.00039 for *TAF1* in iMSNs.

Supplementary Figure 8

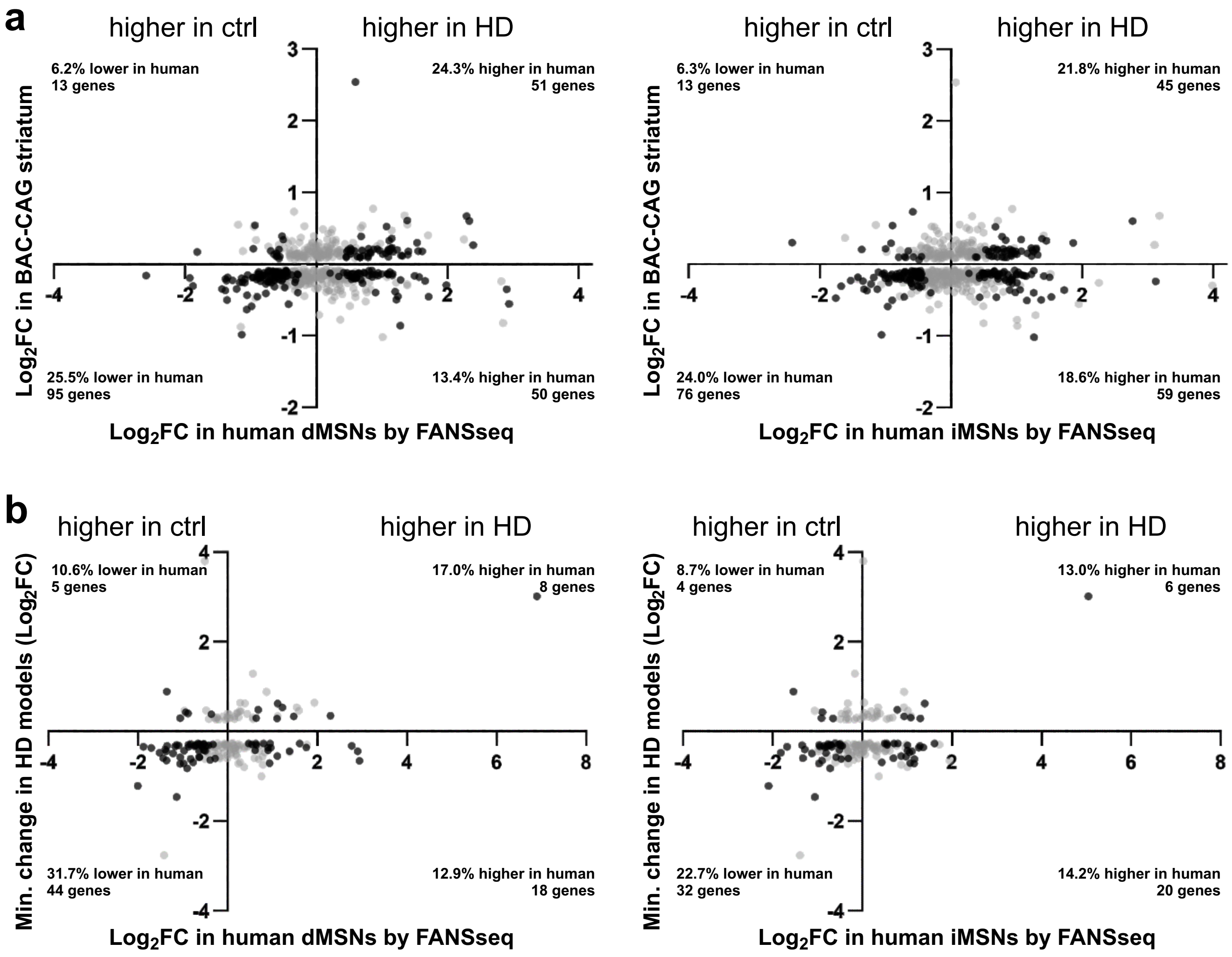

Supplementary Fig. 8. HD-associated transcript level change in human orthologues of (**a**) genes with altered expression in the striatum of BAC-CAG mice (FDR < 0.1)[36] and (**b**) Str266R HD signature genes[37]. The comparisons exclude genes for which none of their annotated TSS positions overlapped with ATAC-seq consensus peaks defined from control or HD donor MSN data. Genes with a significant transcript level change in human FANSseq data are marked with black color (p adj. < 0.05 by DESeq2 after adjusting for multiple comparisons, n=7 individuals with HD and n=8 control individuals).

Supplementary Figure 9

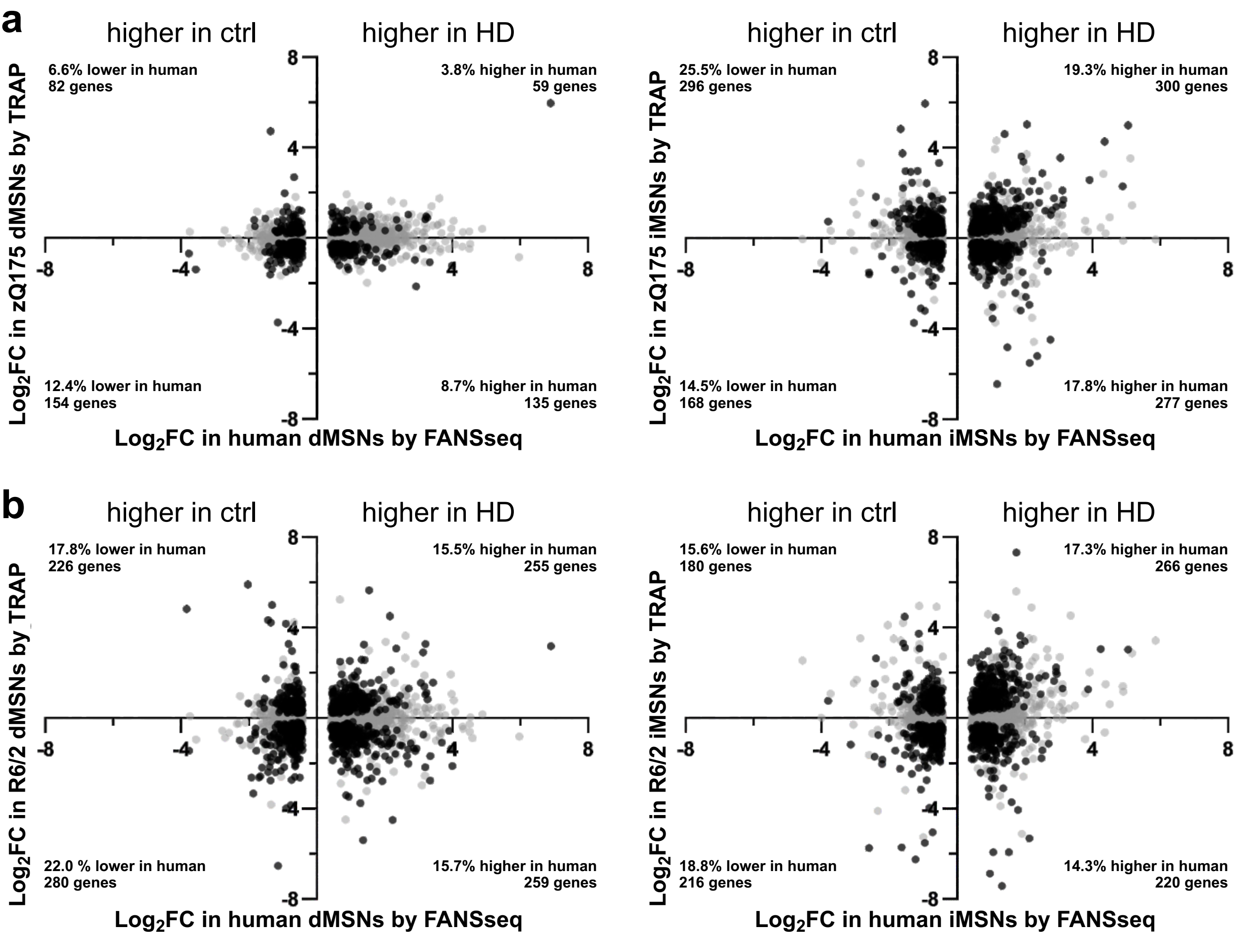

Supplementary Fig. 9. Genes with disease-associated transcript level change in FANSseq data from human iMSN or dMSN (|log2FC|>0.415 and P adj. < 0.05 by DESeq2 after adjusting for multiple comparisons, n=7 individuals with HD and n=8 control individuals) were plotted against the mouse orthologue transcript level change in translational profiling (TRAP) data from iMSNs or dMSNs of (**a**) zQ175 mice (compared to Q20 mice) and or (**b**) R6/2 mice (compared to wt mice)[38]. Genes with a significant transcript level change in the HD mouse model (P adj. < 0.05) are marked with black color. Note that for most of these genes in mouse there is no significant transcript level change according to these criteria.

Supplementary References

1. Caglayan, E., Liu, Y. & Konopka, G. Neuronal ambient RNA contamination causes misinterpreted and masked cell types in brain single-nuclei datasets. *Neuron* **110**, 4043-4056 e5 (2022).
2. Buenrostro, J.D., Giresi, P.G., Zaba, L.C., Chang, H.Y. & Greenleaf, W.J. Transposition of native chromatin for fast and sensitive epigenomic profiling of open chromatin, DNA-binding proteins and nucleosome position. *Nat Methods* **10**, 1213-8 (2013).
3. Ciosi, M. *et al.* A genetic association study of glutamine-encoding DNA sequence structures, somatic CAG expansion, and DNA repair gene variants, with Huntington disease clinical outcomes. *EBioMedicine* **48**, 568-580 (2019).
4. Genetic Modifiers of Huntington's Disease Consortium. Electronic address, g.h.m.h.e. & Genetic Modifiers of Huntington's Disease, C. CAG Repeat Not Polyglutamine Length Determines Timing of Huntington's Disease Onset. *Cell* **178**, 887-900 e14 (2019).
5. Saunders, A. *et al.* Molecular Diversity and Specializations among the Cells of the Adult Mouse Brain. *Cell* **174**, 1015-1030 e16 (2018).
6. McLeay, R.C. & Bailey, T.L. Motif Enrichment Analysis: a unified framework and an evaluation on ChIP data. *BMC Bioinformatics* **11**, 165 (2010).
7. Hayashida, N. *et al.* Heat shock factor 1 ameliorates proteotoxicity in cooperation with the transcription factor NFAT. *EMBO J* **29**, 3459-69 (2010).
8. Wertz, M.H. *et al.* Genome-wide In Vivo CNS Screening Identifies Genes that Modify CNS Neuronal Survival and mHTT Toxicity. *Neuron* **106**, 76-89 e8 (2020).
9. Ito, N. *et al.* Decreased N-TAF1 expression in X-linked dystonia-parkinsonism patient-specific neural stem cells. *Dis Model Mech* **9**, 451-62 (2016).
10. Makino, S. *et al.* Reduced neuron-specific expression of the TAF1 gene is associated with X-linked dystonia-parkinsonism. *Am J Hum Genet* **80**, 393-406 (2007).
11. Campion, L.N. *et al.* Tissue-specific and repeat length-dependent somatic instability of the X-linked dystonia parkinsonism-associated CCCTCT repeat. *Acta Neuropathol Commun* **10**, 49 (2022).
12. Mangiarini, L. *et al.* Instability of highly expanded CAG repeats in mice transgenic for the Huntington's disease mutation. *Nat Genet* **15**, 197-200 (1997).
13. Wheeler, V.C. *et al.* Length-dependent gametic CAG repeat instability in the Huntington's disease knock-in mouse. *Hum Mol Genet* **8**, 115-22 (1999).
14. Silva-Fernandes, A. *et al.* Motor uncoordination and neuropathology in a transgenic mouse model of Machado-Joseph disease lacking intranuclear inclusions and ataxin-3 cleavage products. *Neurobiol Dis* **40**, 163-76 (2010).
15. Switonski, P.M., Szlachcic, W.J., Krzyzosiak, W.J. & Figiel, M. A new humanized ataxin-3 knock-in mouse model combines the genetic features, pathogenesis of neurons and glia and late disease onset of SCA3/MJD. *Neurobiol Dis* **73**, 174-88 (2015).
16. Lopes-Cendes, I. *et al.* Somatic mosaicism in the central nervous system in spinocerebellar ataxia type 1 and Machado-Joseph disease. *Ann Neurol* **40**, 199-206 (1996).
17. Cancel, G. *et al.* Somatic mosaicism of the CAG repeat expansion in spinocerebellar ataxia type 3/Machado-Joseph disease. *Hum Mutat* **11**, 23-7 (1998).
18. Tanaka, F., Ito, Y. & Sobue, G. [Somatic mosaicism of expanded CAG trinucleotide repeat in the neural and nonneural tissues of Machado-Joseph disease (MJD)]. *Nihon Rinsho* **57**, 838-42 (1999).
19. Telenius, H. *et al.* Somatic and gonadal mosaicism of the Huntington disease gene CAG repeat in brain and sperm. *Nat Genet* **6**, 409-14 (1994).
20. Mouro Pinto, R. *et al.* Patterns of CAG repeat instability in the central nervous system and periphery in Huntington's disease and in spinocerebellar ataxia type 1. *Hum Mol Genet* **29**, 2551-2567 (2020).
21. Koeppen, A.H. The Neuropathology of Spinocerebellar Ataxia Type 3/Machado-Joseph Disease. *Adv Exp Med Biol* **1049**, 233-241 (2018).
22. Greco, T.M. *et al.* Dynamics of huntingtin protein interactions in the striatum identifies candidate modifiers of Huntington disease. *Cell Syst* **13**, 304-320 e5 (2022).
23. Wheeler, V.C. *et al.* Mismatch repair gene Msh2 modifies the timing of early disease in Hdh(Q111) striatum. *Hum Mol Genet* **12**, 273-81 (2003).

24. Dragileva, E. *et al.* Intergenerational and striatal CAG repeat instability in Huntington's disease knock-in mice involve different DNA repair genes. *Neurobiol Dis* **33**, 37-47 (2009).
25. Kovalenko, M. *et al.* Msh2 acts in medium-spiny striatal neurons as an enhancer of CAG instability and mutant huntingtin phenotypes in Huntington's disease knock-in mice. *PLoS One* **7**, e44273 (2012).
26. Tome, S. *et al.* MSH3 polymorphisms and protein levels affect CAG repeat instability in Huntington's disease mice. *PLoS Genet* **9**, e1003280 (2013).
27. Wheeler, V.C. & Dion, V. Modifiers of CAG/CTG Repeat Instability: Insights from Mammalian Models. *J Huntingtons Dis* **10**, 123-148 (2021).
28. Gall-Duncan, T. *et al.* Antagonistic roles of canonical and Alternative-RPA in disease-associated tandem CAG repeat instability. *Cell* **186**, 4898-4919 e25 (2023).
29. Tian, L., Gu, L. & Li, G.M. Distinct nucleotide binding/hydrolysis properties and molar ratio of MutSalpha and MutSbeta determine their differential mismatch binding activities. *J Biol Chem* **284**, 11557-62 (2009).
30. Brown, M.W. *et al.* Dynamic DNA binding licenses a repair factor to bypass roadblocks in search of DNA lesions. *Nat Commun* **7**, 10607 (2016).
31. Britton, B.M. *et al.* Exploiting the distinctive properties of the bacterial and human MutS homolog sliding clamps on mismatched DNA. *J Biol Chem* **298**, 102505 (2022).
32. Gupta, S., Gellert, M. & Yang, W. Mechanism of mismatch recognition revealed by human MutSbeta bound to unpaired DNA loops. *Nat Struct Mol Biol* **19**, 72-8 (2011).
33. Owen, B.A. *et al.* (CAG)(n)-hairpin DNA binds to Msh2-Msh3 and changes properties of mismatch recognition. *Nat Struct Mol Biol* **12**, 663-70 (2005).
34. Kim, K.H. *et al.* Genetic and Functional Analyses Point to FAN1 as the Source of Multiple Huntington Disease Modifier Effects. *Am J Hum Genet* **107**, 96-110 (2020).
35. Langfelder, P. *et al.* Integrated genomics and proteomics define huntingtin CAG length-dependent networks in mice. *Nat Neurosci* **19**, 623-33 (2016).
36. Gu, X. *et al.* Uninterrupted CAG repeat drives striatum-selective transcriptionopathy and nuclear pathogenesis in human Huntingtin BAC mice. *Neuron* **110**, 1173-1192 e7 (2022).
37. Obenauer, J.C. *et al.* Expression analysis of Huntington disease mouse models reveals robust striatum disease signatures. *bioRxiv* (2023).
38. Lee, H. *et al.* Cell Type-Specific Transcriptomics Reveals that Mutant Huntingtin Leads to Mitochondrial RNA Release and Neuronal Innate Immune Activation. *Neuron* **107**, 891-908 e8 (2020).
39. Phadte, A.S. *et al.* FAN1 removes triplet repeat extrusions via a PCNA- and RFC-dependent mechanism. *Proc Natl Acad Sci U S A* **120**, e2302103120 (2023).
