# Supplementary material for: Cell-type-specific CAG repeat expansions and toxicity of mutant Huntingtin in human striatum and cerebellum
Source: Nat Genet. 2024 Jan 30;56(3):383–94. doi: 10.1038/s41588-024-01653-6 (PMC10937393; doi:10.1038/s41588-024-01653-6)
Supplement: Supplementary file 2 — Reporting Summary [file 41588_2024_1653_MOESM2_ESM.pdf]

nature portfolio

Corresponding author(s): Nathaniel Heintz

Last updated by author(s): Dec 2, 2023

Reporting Summary

Nature Portfolio wishes to improve the reproducibility of the work that we publish. This form provides structure for consistency and transparency in reporting. For further information on Nature Portfolio policies, see our Editorial Policies and the Editorial Policy Checklist.

Statistics

For all statistical analyses, confirm that the following items are present in the figure legend, table legend, main text, or Methods section.

n/a	Confirmed	
☐	☒	The exact sample size (n) for each experimental group/condition, given as a discrete number and unit of measurement
☐	☒	A statement on whether measurements were taken from distinct samples or whether the same sample was measured repeatedly
☐	☒	The statistical test(s) used AND whether they are one- or two-sided<br>*Only common tests should be described solely by name; describe more complex techniques in the Methods section.*
☒	☐	A description of all covariates tested
☐	☒	A description of any assumptions or corrections, such as tests of normality and adjustment for multiple comparisons
☐	☒	A full description of the statistical parameters including central tendency (e.g. means) or other basic estimates (e.g. regression coefficient) AND variation (e.g. standard deviation) or associated estimates of uncertainty (e.g. confidence intervals)
☐	☒	For null hypothesis testing, the test statistic (e.g. F, t, r) with confidence intervals, effect sizes, degrees of freedom and P value noted<br>*Give P values as exact values whenever suitable.*
☒	☐	For Bayesian analysis, information on the choice of priors and Markov chain Monte Carlo settings
☒	☐	For hierarchical and complex designs, identification of the appropriate level for tests and full reporting of outcomes
☒	☐	Estimates of effect sizes (e.g. Cohen's d, Pearson's r), indicating how they were calculated

Our web collection on statistics for biologists contains articles on many of the points above.

Software and code

Policy information about availability of computer code

Data collection	SONY MA900 Cell Sorter software (ver. 3.0.5) was used for data collection.
Data analysis	Bioconductor package (version 1.4.1), Rsubread (version 1.30.6), rtracklayer package (version 1.40.6), MACS2 (https://github.com/macs3-project/MACS), ChIPseeker package (version 1.28.3), pcaExplorer (2.22.0), DESeq2 (1.36.0), Pheatmap R package (version 1.0.12), Burrows-Wheeler Aligner (0.7.17), clusterProfiler (version 4.4.4, GOSOURCEDATE: 2022-03-10), BioStrings R package (2.66.0), MEME Suite (5.5.4), memes R package (1.6.0), BioRender.com website (date 11-27-2023).

For manuscripts utilizing custom algorithms or software that are central to the research but not yet described in published literature, software must be made available to editors and reviewers. We strongly encourage code deposition in a community repository (e.g. GitHub). See the Nature Portfolio guidelines for submitting code & software for further information.

Data

Policy information about availability of data

All manuscripts must include a data availability statement. This statement should provide the following information, where applicable:

- Accession codes, unique identifiers, or web links for publicly available datasets
- A description of any restrictions on data availability
- For clinical datasets or third party data, please ensure that the statement adheres to our policy

Sequencing datasets generated as part of this study is made publicly available in NCBI GEO under accession GSE227729. Altered expression of mouse genes in the

striatum of BAC-CAG mice (from Gu, X., et al, 2022, Neuron 110, 1173–1192 e1177. doi:10.1016/j.neuron.2022.01.006), the Str266R gene set (Obenauer, J. C., et al, 2023, bioRxiv doi: https://doi.org/10.1101/2022.02.04.479180), list of genes essential for MSN survival in wt mice (data from 'Wertz M., H., et al 2020, Neuron, doi:10.1016/j.neuron.2020.01.004) and TRAP data from zQ175 and R6/2 mice (GEO dataset GSE152058, Lee H., et al 2020, Neuron, doi:10.1016/j.neuron.2020.06.021) have been published before. Sequence and transcript coordinates for human hg38 UCSC genome and gene models were retrieved from the BSgenome.Hsapiens.UCSC.hg38 Bioconductor package (version 1.4.1) and TxDb.Haspiens.UCSC.hg38.knownGene (version 3.4.0) Bioconductor libraries (https://bioconductor.org/packages/release/data/annotation/html/BSgenome.Hsapiens.UCSC.hg38.html). NCBI Refseq hg38 gene annotation (version 109.20211119, https://www.ncbi.nlm.nih.gov/genome/annotation_euk/Homo_sapiens/109.20211119/) was used for annotating ATACseq consensus peaks to transcriptional start sites. Gene Ontology Cellular Compartment (GOCC) terms for enrichment analysis were derived through enrichGO function of clusterProfiler package (version 4.4.4, GOSOURCEDATE: 2022-03-10, https://bioconductor.org/packages/release/bioc/html/clusterProfiler.html). Eukaryote in vivo and eukaryote in vitro databases were accessed through MEME Suite 5.5.4 (https://meme-suite.org/meme/tools/ame)

Research involving human participants, their data, or biological material

Policy information about studies with human participants or human data. See also policy information about sex, gender (identity/presentation), and sexual orientation and race, ethnicity and racism.

Reporting on sex and gender	Huntington's disease afflicts both individuals of both sexes, and both sexes were represented among tissue donors from which material was analyzed. As predominant features of this disease do not manifest in a sex-specific manner, sex-specific analysis was not carried out in this study.
Reporting on race, ethnicity, or other socially relevant groupings	Detailed information on tissue donors is listed in supplementary table 1.
Population characteristics	Detailed information on tissue donors (age, sex, diagnosis, CAP score, post-mortem interval) is listed in supplementary table 1.
Recruitment	*Describe how participants were recruited. Outline any potential self-selection bias or other biases that may be present and how these are likely to impact results.*
Ethics oversight	De-identified tissue samples analyzed in this study were determined to be exempt from Institutional Review Board (IRB) review according to 45 CFR 46.102 (f)

Note that full information on the approval of the study protocol must also be provided in the manuscript.

Field-specific reporting

Please select the one below that is the best fit for your research. If you are not sure, read the appropriate sections before making your selection.

☒ Life sciences ☐ Behavioural & social sciences ☐ Ecological, evolutionary & environmental sciences

For a reference copy of the document with all sections, see nature.com/documents/nr-reporting-summary-flat.pdf

Life sciences study design

All studies must disclose on these points even when the disclosure is negative.

Sample size	Sample size was not predetermined by any calculations, but was rather determined by tissue availability.
Data exclusions	Rigorous quality control was carried out for all datasets produced in the study and a few datasets were excluded from further analysis if transcriptome analysis indicated low RNA quality or the presence of impurities introduced in FACS-sorting step of of the procedure.
Replication	To reduce the possibility of batch effects, samples from control individuals and carriers of mHTT allele were processed in parallel whenever possible. For comparing different cell types, we used samples that were derived from the same set of donors. The main gene expression differences between cell types we have reported were consistent across the two striatal brain regions (processed in independent experiments) - caudate nucleus and putamen (as documented in the figures). Almost all of the disease-associated changes we have highlighted were consistent across the two striatal projection neuron subtypes (dMSNs and iMSNs). Statistical analysis was done on data derived from independent experiments (the number of replicates is described in the figure legends).
Randomization	Samples were allocated into experimental groups based on their clinical diagnosis (control donors, HD donors, SCA3 donors): clinical symptoms and the presence of causal mutation in HTT or ATXN3 genes. The tissue donors were selected so that inter-group differences in age and sex would be minimal.
Blinding	Blinding was not relevant to the study as Next-Generation Sequencing data analysis was carried out in an automated manner using identical parameters and settings for all samples. Quantification of band intensities was done using analysis software, making the quantification process more consistent and less prone to experimenter bias.

Reporting for specific materials, systems and methods

We require information from authors about some types of materials, experimental systems and methods used in many studies. Here, indicate whether each material, system or method listed is relevant to your study. If you are not sure if a list item applies to your research, read the appropriate section before selecting a response.

Materials & experimental systems

n/a	Involved in the study
☐	☒ Antibodies
☐	☒ Eukaryotic cell lines
☒	☐ Palaeontology and archaeology
☒	☐ Animals and other organisms
☒	☐ Clinical data
☒	☐ Dual use research of concern
☒	☐ Plants

Methods

n/a	Involved in the study
☒	☐ ChIP-seq
☒	☐ Flow cytometry
☒	☐ MRI-based neuroimaging

Antibodies

Antibodies used	Antibodies and PrimeFlow probes<br>anti-IRF8-PE Thermo Fisher Scientific Cat# 12-9852-82, RRID:AB_2572742<br>anti-EAAT1 Santa Cruz Biotechnology Cat# sc-515839<br>anti-NeuN-Alexa Fluor 594 Abcam Cat# ab207279<br>anti-NeuN-Alexa Fluor 647 Abcam Cat# ab190565, RRID:AB_2732785<br>anti-Olig2 R&D systems Cat# AF2418, RRID:AB_2157554<br>anti-Mouse IgG Alexa Fluor 488 Thermo Fisher Scientific Cat# A-21202, RRID:AB_141607<br>anti-Goat IgG Alexa Fluor 647 Thermo Fisher Scientific Cat# A-21447, RRID:AB_141844<br>anti-ITPR1-Alexa Fluor 488 Santa Cruz Biotechnology Cat# sc-271197, RRID:AB_10610775<br>anti-Histone H3 Abcam Cat# ab1791, RRID:AB_302613<br>anti-MSH2 BD Biosciences Cat# 556349, RRID:AB_396378<br>anti-MSH3 BD Biosciences Cat# 611390, RRID:AB_398912<br>IRDye 680LT Donkey anti-Rabbit IgG LI-COR Biosciences Cat# 926-68023, RRID:AB_10706167<br>IRDye 800CW Goat anti-Mouse IgG LI-COR Biosciences Cat# 926-32210, RRID:AB_621842<br>DRD1 PrimeFlow probe Alexa Fluor 647 Thermo Fisher Scientific Cat# VA1-3002351-PF<br>DRD2 PrimeFlow probe Alexa Fluor 488 Thermo Fisher Scientific Cat# VA4-3083767-PF<br>PPP1R1B PrimeFlow probe Alexa Fluor 568 Thermo Fisher Scientific Cat# VA10-3266354-PF<br>TAC3 PrimeFlow probe Alexa Fluor 647 Thermo Fisher Scientific Cat# VA1-16603-PF<br>ETV1 PrimeFlow probe Alexa Fluor 488 Thermo Fisher Scientific Cat# VA4-3083818-PF<br>SST PrimeFlow probe Alexa Fluor 568 Thermo Fisher Scientific Cat# VA10-3252595-PF<br>TRPC3 PrimeFlow probe Alexa Fluor 647 Thermo Fisher Scientific Cat# VA1-3004835-PF<br>COL6A6 PrimeFlow probe Alexa Fluor 647 Thermo Fisher Scientific Cat# VA1-3014134-PF<br>CA8 PrimeFlow probe Alexa Fluor 647 Thermo Fisher Scientific Cat# VA1-3001892-PF
Validation	The specificity of all antibodies and Primeflow probes used to label nuclei was validated by analyzing the nuclear transcriptome of labeled nuclei and verifying the presence or absence of transcripts of marker genes known to be expressed by only by certain cell types.

Eukaryotic cell lines

Policy information about cell lines and Sex and Gender in Research

Cell line source(s)	Sf9 cells were purchased from ATCC.<br>https://www.atcc.org/products/crl-1711
Authentication	The authentication was done by ATCC, and not redone as the cells do look quite different than human cells lines and are grown in specific media (Grace's media at 27C).
Mycoplasma contamination	There was no mycoplasma contamination.
Commonly misidentified lines<br>(See ICLAC register)	none.
