# Supplementary material for: Cell-type-specific CAG repeat expansions and toxicity of mutant Huntingtin in human striatum and cerebellum
Source: Nat Genet. 2024 Jan 30;56(3):383–94. doi: 10.1038/s41588-024-01653-6 (PMC10937393; doi:10.1038/s41588-024-01653-6)
Supplement: Supplementary file 6 — Unprocessed images of western blots. [file 41588_2024_1653_MOESM6_ESM.pdf]

Source Data for Figure 5

Source data for anti-MSH2 western blot quantification

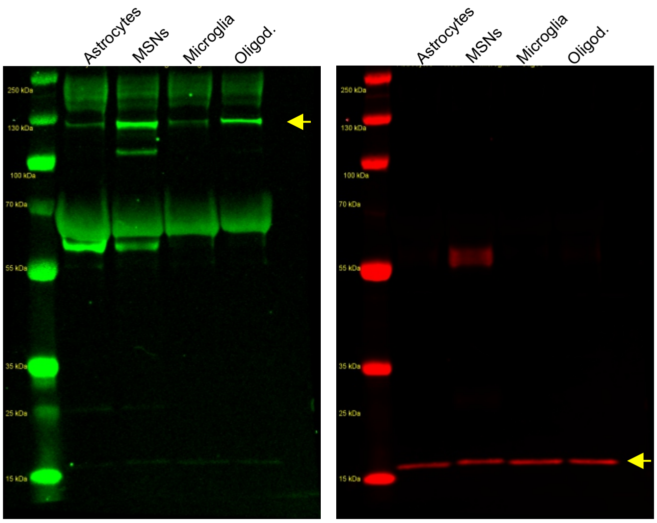

Mm anti-MSH2 Rb anti-H3

donor HBBS, Putamen

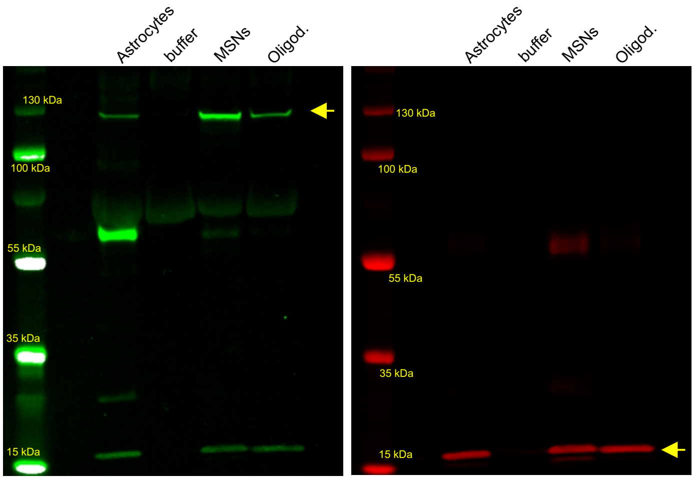

Mm anti-MSH2 Rb anti-H3

donor HctYZ, Putamen

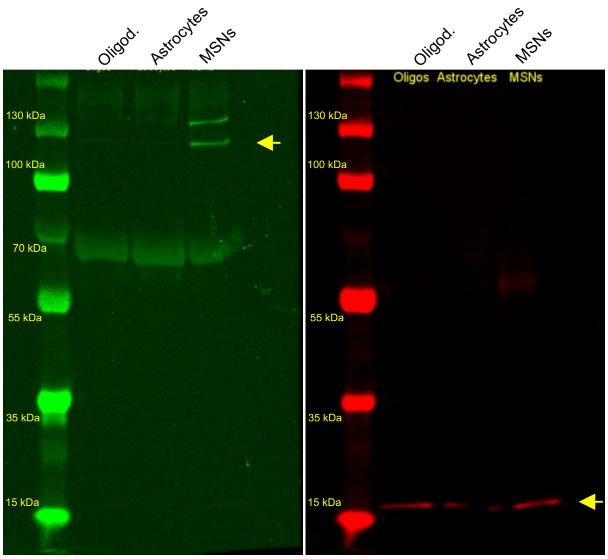

Mm anti-MSH2 Rb anti-H3

donor HctZM, Putamen

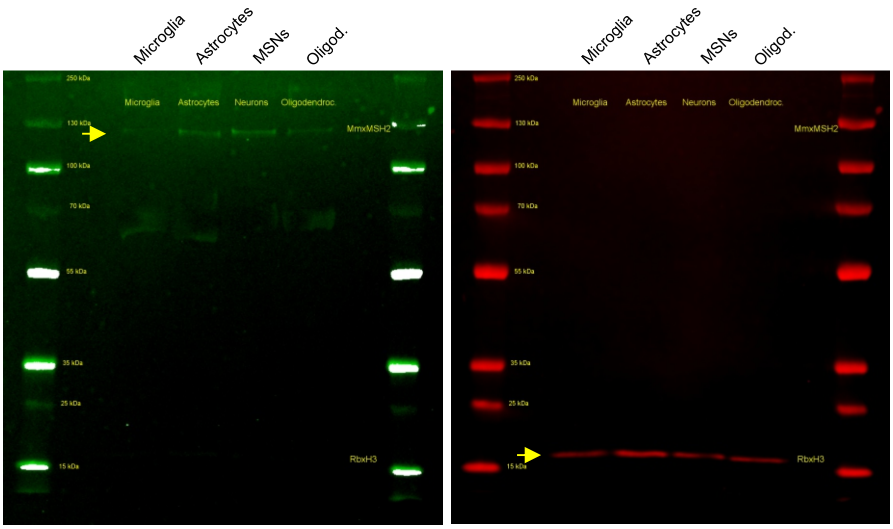

Mm anti-MSH2 Rb anti-H3

donor Hct16HCG, Putamen

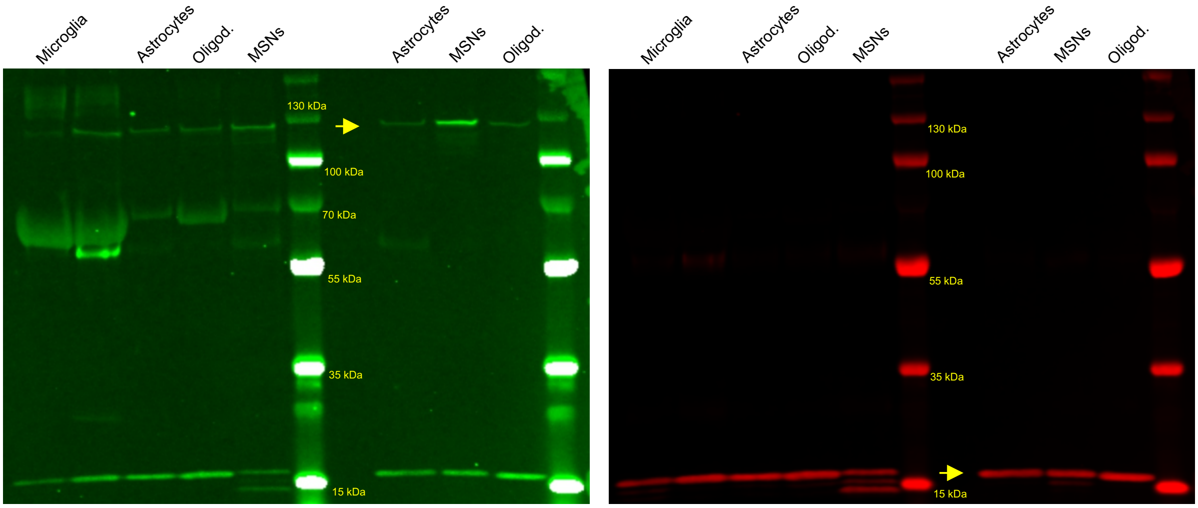

Mm anti-MSH2 Rb anti-H3

donor BEB18076, Put. donor Hct16HCH, Put. donor BEB18076, Put. donor Hct16HCH, Put.

Source Data for Figure 5. Arrows point to MSH2 and H3 signal on blots probed with anti-MSH2 and anti-H3, respectively. Additional bands likely represent BSA present in the buffer (below 70 kDa) and heavy chain of antibodies used for staining nuclei for FANS (around 60 kDa).

Source Data for Figure 5
Source data for anti-MSH3 western blot quantification

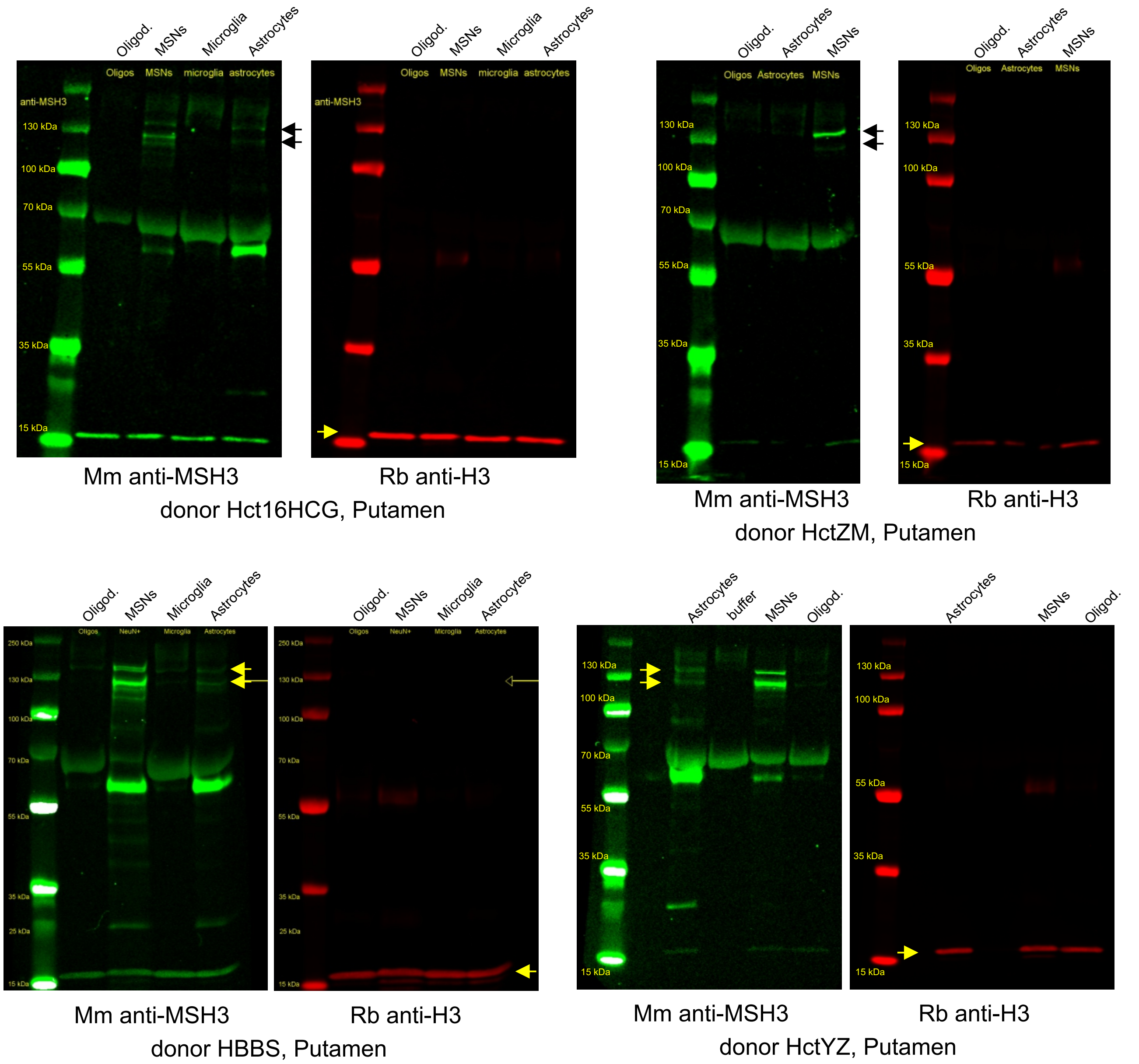

Source Data for Figure 5. Arrows point to MSH3 and H3 signal on blots probed with anti-MSH3 and anti-H3, respectively. Additional bands likely represent BSA present in the buffer (below 70 kDa) and heavy chain of antibodies used for staining nuclei for FANS (around 60 kDa).
